# Knockdown of Sec16 causes early lethality and defective deposition of the protein Rp30 in the eggshell of the vector *Rhodnius prolixus*

**DOI:** 10.3389/fcell.2024.1332894

**Published:** 2024-04-22

**Authors:** Thamara Rios, Larissa Bomfim, Jéssica Pereira, Kildare Miranda, David Majerowicz, Attilio Pane, Isabela Ramos

**Affiliations:** ^1^ Instituto de Bioquímica Médica Leopoldo de Meis, Universidade Federal do Rio de Janeiro, Rio de Janeiro, Brazil; ^2^ Instituto de Biofísica Carlos Chagas Filho, Universidade Federal do Rio de Janeiro, Rio de Janeiro, Brazil; ^3^ Departamento de Biotecnologia Farmacêutica, Faculdade de Farmácia, Universidade Federal do Rio de Janeiro, Rio de Janeiro, Brazil; ^4^ Programa de Pós-Graduação em Biociências, Universidade do Estado do Rio de Janeiro, Rio de Janeiro, Brazil; ^5^ Instituto Nacional de Ciência e Tecnologia em Entomologia Molecular, Rio de Janeiro, Brazil; ^6^ Instituto de Ciências Biomédicas, Universidade Federal do Rio de Janeiro, Rio de Janeiro, Brazil

**Keywords:** oogenesis, follicle cells, chorion, secretory pathway, Sec16

## Abstract

In nearly every species of insect, embryonic development takes place outside of the mother’s body and is entirely dependent on the elements that the mother had previously stored within the eggs. It is well known that the follicle cells (FCs) synthesize the eggshell (chorion) components during the process of choriogenesis, the final step of oogenesis before fertilization. These cells have developed a specialization in the massive production of chorion proteins, which are essential for the protection and survival of the embryo. Here, we investigate the function of Sec16, a protein crucial for the endoplasmic reticulum (ER) to Golgi traffic, in the oocyte development in the insect *Rhodnius prolixus*. We discovered that Sec16 is strongly expressed in vitellogenic females’ ovaries, particularly in the choriogenic oocyte and it is mainly associated with the FCs. Silencing of Sec16 by RNAi caused a sharp decline in oviposition rates, F1 viability, and longevity in adult females. In the FCs, genes involved in the unfolded protein response (UPR), the ubiquitin-proteasome system (UPS), and autophagy were massively upregulated, whereas the mRNAs of Rp30 and Rp45—which code for the two major chorion proteins - were downregulated as a result of Sec16 silencing, indicating general proteostasis disturbance. As a result, the outer surface ultrastructure of Sec16-silenced chorions was altered, with decreased thickness, dityrosine crosslinking, sulfur signals, and lower amounts of the chorion protein Rp30. These findings collectively demonstrate the critical role Sec16 plays in the proper functioning of the FCs, which impacts the synthesis and deposition of particular components of the chorion as well as the overall reproduction of this vector.

## Introduction

In oviparous species, such as insects, the embryo’s viability is reliant on the protection provided by the egg and all its previously accumulated resources. The mechanism that precedes egg laying is called oogenesis, which can be divided into two main stages: vitellogenesis and choriogenesis. The first one is mainly characterized by the rapid growth of the oocyte due to the massive yolk uptake, resulting in the accumulation of reserve macromolecules necessary for embryonic development (Oliveira et al., 1986; Raikhel and Dhadialla, 1992; Ramos et al., 2022). At the end of vitellogenesis, the process of choriogenesis begins. In this step, the components that will form the chorion (eggshell), an extracellular matrix that has essential functions for embryo survival, such as the regulation of gas exchange and protection against drying and parasite invasion, will be synthesized and deposited over the surface of the oocyte (Beament, 1946; Huebner and Anderson, 1972; Hinton, 1981; Zrubek and Woods, 2006; Bomfim et al., 2017). The follicle cells (FCs) are intimately associated with the growing oocytes forming a single-layered tissue that envelops the oocytes and undergoes a high metabolic challenge to sustain the demand for synthesis of the chorion components (Huebner and Anderson, 1972; Wu et al., 2008; Medeiros et al., 2011). After synthesizing within the FCs, the multiple chorion components must be exported and deposited on the oocyte’s surface presumably through the canonical secretory pathway. The coat protein complex II (COPII) acts in the transport of vesicles that carry the content produced in the endoplasmic reticulum (ER) to the Golgi apparatus (Bonifacino and Glick, 2004; Kirchhausen, 2020). COPII vesicles bud at ribosome-free sites in the ER called ER exit sites (ERES), and its coat is formed from the recruitment of a set of 7 conserved proteins: Sar1, Sec12, Sec23, Sec24, Sec13, Sec31, and Sec16 (Barlowe et al., 1994; Lee et al., 2004; Spang, 2009; Bisnett et al., 2021). Secretory 16 (Sec16) is a scaffold protein that localizes to the ERES and interacts with many components of the COPII coating, where it acts as a recruiter and provides structural support for the other COPII proteins potentiating vesicle assembly and budding (Supek et al., 2002; Connerly et al., 2005; Watson et al., 2006; Ivan et al., 2008; Montegna et al., 2012; Tang, 2017). Interestingly, Sec16 has also been described as a partner of the autophagy-related gene 1 (ATG1) in the secretory pathway (Joo et al., 2016).

In insects, studies on choriogenesis primarily concentrate on the biochemical characterization of chorion proteins (Fakhouri et al., 2006) and the programmed gene-specific transcriptional activation of chorion genes, particularly in *Drosophila melanogaster* and *Bombyx mori* (Kafatos et al., 1995; Tootle et al., 2011; Velentzas et al., 2018). Evidence suggests that the chorion morphology and protein composition are highly species-specific (Papantonis et al., 2015). Aspects of the chorion ultrastructure and permeability properties in *Rhodnius prolixus* have been studied in the past (Beament, 1946; Dias et al., 2013; Bomfim et al., 2017), and the specific chorion proteins Rp30 and Rp45, the latter of which is linked to antifungal activity, have also been identified and described (Bouts et al., 2007).

*R. prolixus* is a hemipteran insect and one of the main vectors of Chagas disease. As a significant neglected tropical disease, vector control is vital to prevent this illness (WHO, 2022). Our research group previously reported that the silencing of ATG1 in the FCs activated ER stress and produced eggs with deformed chorion ultrastructure, an observation that was demonstrated to be phenocopied by the silencing of Sec16 (Bomfim and Ramos, 2020). Here, we further explore the role of Sec16 in *R. prolixus* physiology and chorion biogenesis. We mined the *de novo* assembly RNAseq transcripts (Coelho et al., 2021) to identify the full open reading frame of the *R. prolixus* Sec16, which was scattered in different genome contigs. Phylogenetic analysis revealed that *R. prolixus* contains one single isoform of the Sec16 gene. RNAi-silencing of Sec16 resulted in early lethality and decreased oviposition rates. In the FCs, we found that silencing of Sec16 triggers the upregulation of autophagy, ubiquitin proteasome system (UPS), and unfolded-protein response (UPR) effectors. In contrast, the main chorion proteins Rp30 and Rp45 presented reduced expression levels. Accordingly, the chorions from silenced insects showed altered thickness and outer surface ultrastructure, reduced protein crosslinking, sulfur signals, and lower levels of the chorion protein Rp30. These factors are all likely to be the cause of the observed embryonic lethality.

These findings show that Sec16 is essential for the FCs to operate correctly, affecting the chorion synthesis and deposition. As the coordination of the process of protein synthesis and transport for the chorion biogenesis must be performed with extreme rigor, we discuss how the identification of novel molecular targets that interfere with choriogenesis has the potential to become an important strategy to be applied in the creation of cutting-edge tools for vector population control.

## Materials and methods

### Insects and eggs

Adult females and eggs were maintained in a controlled insectary at a temperature of 28°C, with a photoperiod of 12 h light/dark, and relative humidity of around 70%–80%. At 21 days intervals, the adult insects were fed with rabbit blood, following a protocol approved by the Ethics Committee on the Use of Animals (CEUA-UFRJ), registered under process number 01200.001568/2013-87 and order number 155/13 in the National Council for the Control of Animal Experimentation (CONCEA).

### Phylogenetic analyses

In addition to the *R. prolixus* sequence*,* we explored the genomes of *Acyrthosiphon pisum, Aedes aegypti, Aedes albopictus, Anopheles gambiae, Apis mellifera, Bemisia tabaci, B. mori, Cimex lectularius, Culex quinquefasciatus, D. melanogaster*, *Heliconius melpomene, Lutzomyia longipalpis, Manduca sexta, Nilaparvata lugens, Pediculus humanus corporis, Pogonomyrmex barbatus, Schistocerca americana, Tribolium castaneum, and Zootermopsis nevadensis.* All proteins containing the Pfam domain named Sec16_C (PF12931) were obtained from the EnsemblMetazoa database (Yates et al., 2022) using the BioMart tool (Kinsella et al., 2011). The sequence of collembolan *Orchesella cincta* was included as an external group. Using the MEGA X software, the primary sequences were aligned using the MUSCLE tool (Edgar, 2004), and the phylogenetic analysis was performed by the maximum likelihood method (Felsenstein, 1981) with 1000 bootstrap repetitions.

### Extraction of total RNA and cDNA synthesis

For total RNA extraction, different organs of vitellogenic females were dissected 7 days after blood feeding. All dissections were performed at 7 days after the blood meal. In our experience, this is the first time point when the vitellogenic ovaries terminate the production of the first batch of chorionated oocytes for most of the insects. Thus, we know that the first cycle of oocyte development is terminated, and the ovary samples often include developing and fully grown oocytes.

Furthermore, the various stages of oogenesis (tropharium, oocytes, and their respective FCs) were dissected separately. The samples were homogenized using a plastic potter in 500 μL of Trizol reagent, and the RNA was extracted. The pellet was resuspended in 20 μL of H_2_O DEPC and absorbance at 260 nm was measured by spectrophotometry in a NanoDrop (Thermo Scientific). After DNase I (Invitrogen) treatment, 1 µg of total RNA was utilized to reverse transcription reaction using the High-Capacity cDNA Reverse Transcription Kit (Applied Biosystems), according to the manufacturer’s protocol.

### Dissection of the follicle cells

Vitellogenic and choriogenic follicles are dissected 7 days after the blood meal and immersed in ice cold PBS. Using fine forceps, dissecting scissors and a stereomicroscope, a longitudinal tear is made in each follicle, leading to the release of oocyte contents from the FCs tissue. The remaining tissue is then transferred to a new petri dish to be washed in ice cold PBS and used for the different analyses.

### PCR and quantitative PCR (qPCR)

PCR reactions were performed in a thermocycler using specific primers for *R. prolixus* Sec16, designed using the OligoAnalyzer tool from the IDT - Integrated DNA Technologies website (https://www.idtdna.com/) to amplify a 151 bp fragment, as previously described (Bomfim and Ramos, 2020). The PCR product was further tested and visualized in a 2% agarose gel. qPCR reactions were performed in a StepOne Real-Time PCR System thermocycler (Applied Biosystems), using SYBR Green PCR Master Kit (Applied Biosystems) and with the following program for the amplification reaction: an initial denaturation step at 95°C for 10 min, 40 cycles at 95°C for 15 s and 60°C for 1 min. The samples were obtained from a pool of 2–3 wild-type or injected females for each biological replicate. Relative expressions were obtained using the mean of Ct duplicates of the endogenous Rp18s (RPRC017412) and experimental genes. The 18s gene was used as the single reference gene because there is previous evidence of its invariable expression under our experimental conditions (Bomfim and Ramos, 2020; Vieira et al., 2021; Pereira et al., 2022). The invariable expression of 18s specifically after the silencing of Sec16 is shown in Supplementary Figure S1. All calculations were made through 2^−dCt^, all according with the minimum information for publication of quantitative RT-qPCR experiments (MIQE) guidelines (Bustin et al., 2009). All the specific primer sequences are described in Supplementary Table S1.

### RNAi silencing

dsRNAs were synthesized by MEGAScript RNAi Kit (Ambion Inc) using primers for specific gene amplification with the T7 promoter sequence. Two dsRNAs were designed to target regions of 768 bp and 355 bp from the Sec16 gene (Bomfim and Ramos, 2020). The MalE gene from *Escherichia coli*, which encodes a maltose-binding protein, was used as an injection control since this gene is absent in *R. prolixus*. Unfed adult females were injected using a 10 μL Hamilton syringe 3 days before blood meal with control and experimental dsRNAs directly into the hemolymph, with 1 μL of dsMal at a concentration of 1 μg/μL and 1 μL of a mix containing both dsSec16, each of them with a concentration of 0.5 μg/μL. The knockdown efficiency was posteriorly confirmed by qPCR 7 days after blood meal.

### Main aspects of insect physiology

After the dsRNA injection described above, the insects were individualized and weighed before and after the blood meal at different time points. Eggs laid were collected on the same days the animals were weighed for analysis of oviposition, and, approximately 15 days later, the hatched nymphs were counted. Finally, the longevity of females was measured by observing the day each insect died.

### Hemolymph extraction and SDS-PAGE

The hemolymph was extracted from control and silenced females 7 days after the blood meal, as previously described (Masuda and Oliveira, 1985). Approximately 10 µL of hemolymph per female was obtained by cutting 1 of the insects’ legs and applying gentle pressure to the abdomen. The hemolymph was collected using a 10 µL pipette plastic tip. Once collected, the hemolymph was diluted 2 × in HEPES buffer 50 mM pH 7.4 containing protease inhibitors (aprotinin 0.3 µM, leupeptin 1 μg/μL, pepstatin 1 μg/μL, PMSF 100 μM, and EDTA 1 mM), and approximately 8 mg of phenylthiourea. The equivalent of 1 µL of hemolymph was loaded in each lane of a 10% SDS-PAGE and stained with silver nitrate (Merril et al., 1981). Densitometry was performed using ImageJ software. Each biological replicate was prepared using the hemolymph of 1 individual.

### Oocyte homogenates and SDS-PAGE

Control and silenced choriogenic oocytes were dissected 7 days post blood meal. The homogenates were prepared by immersing the oocytes in 50 μL of HEPES buffer 50 mM pH 7.4 containing protease inhibitors (aprotinin 0.3 μM, leupeptin 1 μg/μL, pepstatin 1 μg/μL, PMSF 100 μM and EDTA 1 mM) and then disrupting (breaking) their chorions using a plastic pestle. After a few seconds, the chorion fragments generated decant in the tube, and the supernatant, containing the solubilized oocyte proteins, is transferred to a new tube and used for protein quantifications and SDS-PAGEs. The equivalent of 1-tenth of the volume of the oocyte was loaded in a 10% SDS-PAGE and stained with silver nitrate. Densitometry was performed using ImageJ software. For each biological replicate, the samples were prepared using a pool of 2 oocytes laid by 1 individual.

### Determination of protein content

The total amount of protein levels in the hemolymph and oocytes were measured using the Lowry (Folin) method, with 1–7 µg of BSA serving as the standard control (Lowry et al., 1951). Measurements were performed in an E-MAX Plus microplate reader (Molecular Devices) using SoftMax Pro 7.0 as software. For each biological replicate, hemolymph samples were obtained from 1 individual. For oocyte homogenates, a pool of 2 choriogenic oocytes was obtained from 1 individual per biological replicate.

### Light microscopy

Operculum of recently dissected choriogenic oocytes were carefully detached using a sharp razor blade under the stereomicroscope. The operculum-free oocytes were fixed by immersion in 4% freshly prepared formaldehyde and 2.5% glutaraldehyde (Grade I) in 0.1 M cacodylate buffer, pH 7.3, for at least 24 h at room temperature. Samples were washed 3 times for 5 min in the same buffer, infiltrated in increasing sucrose concentrations (5%, 10%, 15%, and 20%) as a cryoprotectant, and embedded in increasing concentrations (25%, 50%, 75%, and 100%) of OCT compound medium (Tissue-TEK), for 24 h for each of the concentrations. Once infiltrated in pure OCT, 15–30 μm transversal sections were obtained in a cryostat. The slides were mounted in glycerol 70%, followed by observation in a Zeiss Axio Imager D2 microscope equipped with a Zeiss Axio Cam MRc 5 digital camera operated in a differential interferential contrast (DIC) mode.

### Scanning electron microscopy

0–72 h laid eggs were carefully collected and fixed by immersion in 2.5% glutaraldehyde (Grade I) and 4% freshly prepared formaldehyde in 0.1 M cacodylate buffer, pH 7.3. Samples were washed in cacodylate buffer, dehydrated in an ethanol series (15%, 30%, 50%, 70%, 90%, and 100%), critical point dried and coated with a 10 nm layer of gold. Models were observed in an FEI Quanta 250 field emission scanning electron microscope operating at 15 kV. To measure the chorion thickness, the chorions were carefully transversally sectioned using a fine forceps and a sharp blade under the stereomicroscope. The chorion fragments were then vertically placed in the SEM stubs, so that a side view of the chorions would face the electron beam and their thickness could be directly visualized. The mean thickness was calculated using 3 measurements taken from different regions of the chorion. Images of 4 different eggs per group were obtained.

### Dityrosine chorion fluorescence

0–72 h eggs were photographed using the Zeiss Axiozoom V.16 stereomicroscope using the DAPI filter set as previously described (Dias et al., 2013). For the fluorescence quantification, ImageJ software was used to analyze images of 5 eggs from each group, where the approximate chorion fluorescence (CF) was calculated using the following formula: CF = integrated density - (area of selected egg x mean fluorescence of background readings).

### X-ray microanalysis by dispersive energy and elemental mapping

0–72 h eggs laid by females injected with dsMal and dsSec16 mix were carefully collected and examined directly under a FEI Quanta 250 scanning electron microscope operating at 12.5 kV. X-rays were collected by a silicon-lithium detector for 100 s, in a range of 0–5 keV of energy, to capture the spectra and elemental mapping. The relative concentrations of the sulfur element were measured by the semi-quantitative Cliff-Lorimer method as previously described (Miranda et al., 2004).

### Chorion protein extraction and SDS-PAGE

0–72 h eggs were collected and carefully washed to remove the internal content in 0.01 M Tris/HCl, pH 8.4 several times. Chorions from 5 control and Sec16-silenced eggs were subjected to chemical and mechanical protein extraction as previously described with some modifications (Bouts, et al., 2007). Briefly, the washed and dried chorions on filter paper were homogenized in 250 μL of denaturing extraction buffer containing 8 M urea, 360 mM Tris/HCl pH 8.4, and 30 mM dithiothreitol (DTT) using a glass/Teflon potter Elvehjem homogenizer. After homogenization, the samples were centrifuged for 10 min at 12,000 g at 4°C, and the supernatant was collected and stored at −20° until further use. The samples were loaded in a 10% SDS-PAGE, followed by silver nitrate staining. Densitometry was performed using ImageJ software.

### Statistics

The results were submitted to the One-Way ANOVA, Two-Way ANOVA (both followed by Tukey’s Multiple Comparison test), Student's t-test or Log-Rank (Mantel-Cox) test, all of them using the GraphPad Prism 8 software. Differences were considered significant at *p* < 0.05.

## Results

### The single isoform of the *R. prolixus* Sec16 gene is highly expressed in the ovary, particularly in the follicle cells of developing oocytes

Initially, we searched for the Sec16 gene in the genome of *R. prolixus* available on the VectorBase platform (Rpro C3.4) (Mesquita et al., 2015). Two sequences were annotated: RPRC002699, named RpSec16_1, and RPRC002702, named RpSec16_2. Although the putative protein encoded by RPRC002699 shares high sequence similarity with orthologs from distantly related insect species, it was immediately apparent the *R. prolixus* protein was likely to be incomplete. We noticed that the genomic region harboring RPRC002699 displays several sequencing gaps, suggesting that the remaining part of the gene might be scattered in other genomic contigs. To retrieve the full-length ORF of RpSec16, we performed *de novo* transcriptome assembly with RNAseq datasets generated from previtellogenic stages of oogenesis and mature eggs (Coelho et al., 2021). After trimming and quality checking, the reads were assembled using Trinity with default parameters. We then used the RPRC002699 and RPRC002702 sequences to blast against the new list of Trinity-assembled transcripts. This approach allowed us to identify a 7230 nt long transcript in the previtellogenic datasets corresponding to the RpSec16 gene. Interestingly, the RpSec16 transcript encompasses not only the RPRC002702 and RPRC002699 but also the RPRC002689 transcription unit.

**FIGURE 1:**
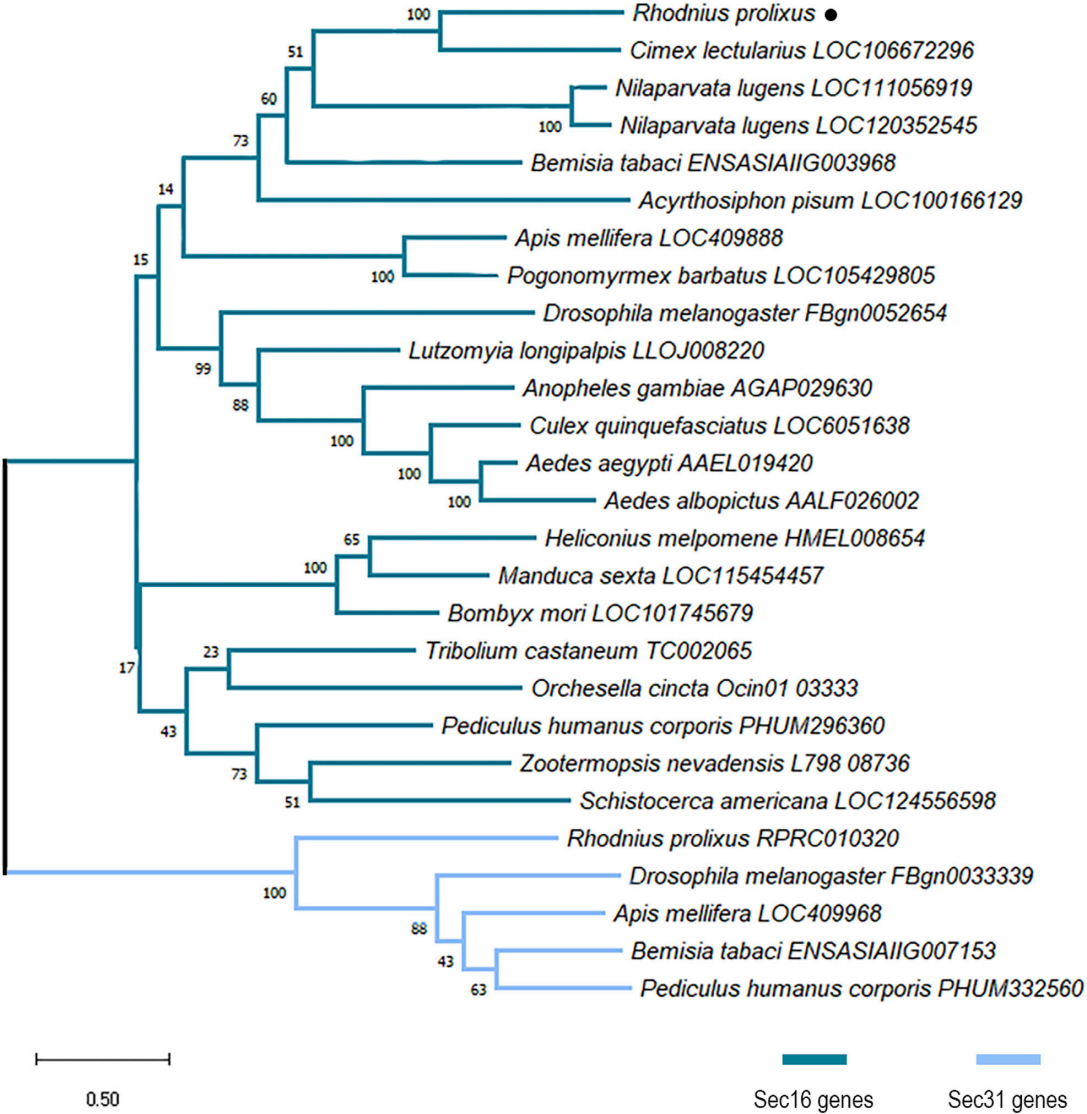
Maximum likelihood phylogenetic analysis of proteins with PF12931 domain across species. Sequences were aligned using the MUSCLE tool and the phylogenetic tree reconstruction was made using the maximum likelihood method. The tree was designed with 1000 replicates of rapid bootstrap statistics. Dark blue represents Sec16 proteins from different species. Light blue represents Sec31 proteins from different species. *R. prolixus* Sec16 is dot-marked.

The structure of the Trinity contig and the consensus sequence of the putative full ORF of *R. prolixus* Sec16 (RpSec16) was confirmed by PCR amplification with nine different pairs of primers designed to cover the entire 7173 bp sequence (Supplementary Figures S2A–C and Supplementary Document S1). Multiple amino acid sequence alignment of RpSec16 with orthologs from other organisms corroborates these findings as we have seen that phylogenetically distant species have a specific rate of identity and similarity (>30%), with *R. prolixus* being closer to the hemipteran insect *C. lectularius* (68% and 53% similarity and identity, respectively) (Supplementary Figure S2D). Furthermore, phylogenetic analysis confirmed that the final RpSec16 sequence is indeed the homolog of Sec16 from *R. prolixus* (Figure 1). Interestingly, while all the insects analyzed have an ortholog of Sec16, *N. lugens* presented 2 genes with evidence of a recent duplication. On the other hand, only a few insects analyzed have an ortholog of Sec31, including *R. prolixus* (RPRC010320) (Figure 1).

**FIGURE 2:**
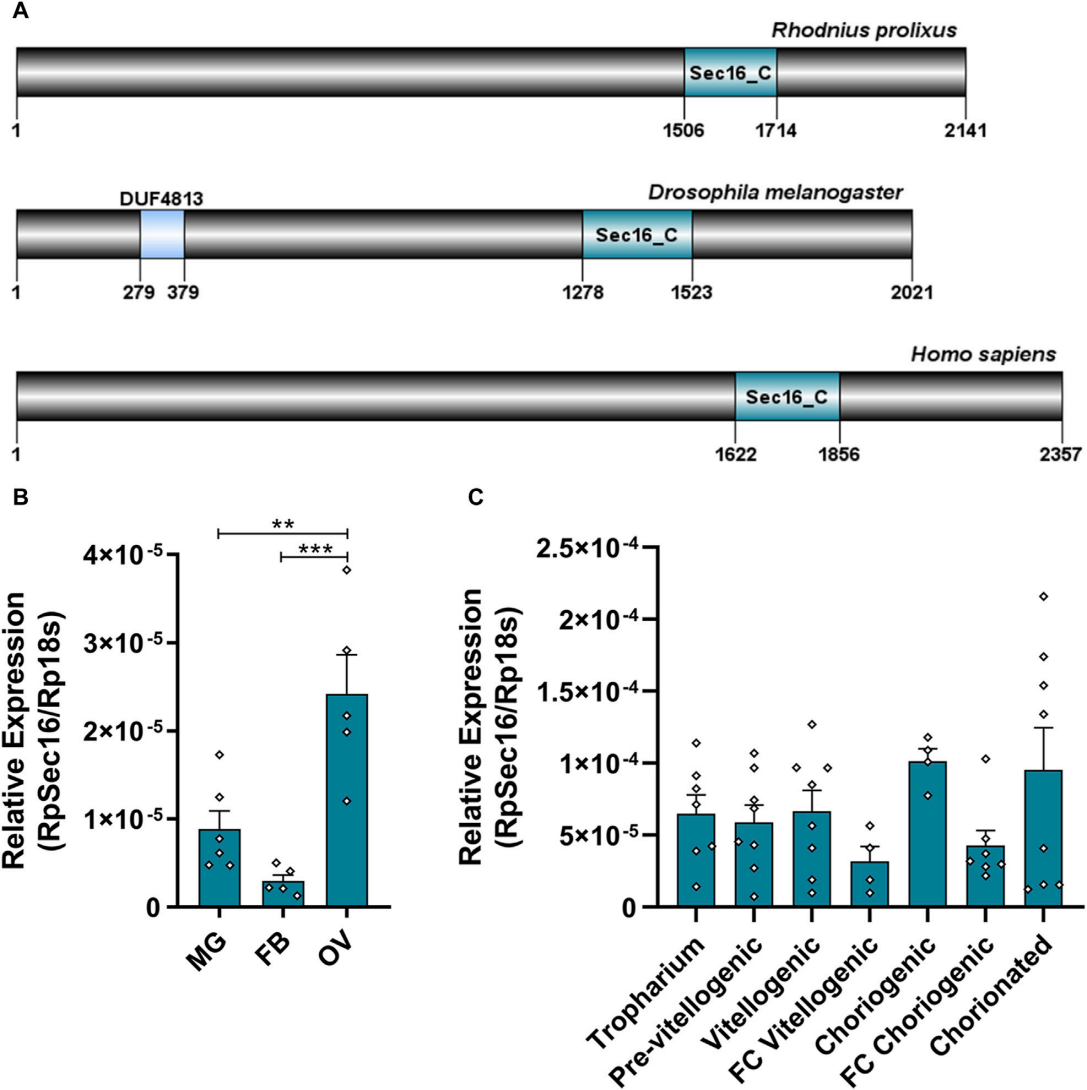
Sec16 is highly expressed in the ovary and is present in all stages of oogenesis in vitellogenic females. **(A)** Representative scheme of the Sec16 protein from *R. prolixus*, *D. melanogaster,* and *H. sapiens* and their conserved domains Sec16_C (pfam12931), the binding domain of the Sec23 protein. **(B)** RT-qPCR showing the relative expression levels of Sec16 in different organs of the adult female. MG: Midgut; FB: Fat body; OV: Ovary. (*n* = 5–6). One-way ANOVA. **(C)** RT-qPCR showing the relative expression levels of Sec16 throughout all stages of oogenesis. (*n* = 4–8). One-way ANOVA. Graphs show mean ± SEM. **p < 0.01, ***p < 0.001.

The RpSec16 gene encodes a putative 2391 amino acids long protein. It was possible to identify the conserved domain Sec16_C (pfam12931), characteristic of the protein in question (Figure 2A). RpSec16 mRNA was found via RT-qPCR to be expressed in the ovary, midgut, and fat body of adult vitellogenic females, with the ovary exhibiting the highest levels of expression, on average, 2.7x and 8.1x higher than the midgut and fat body, respectively (Figure 2B). In the ovaries, after the dissection of the tropharium and all stages of oocyte development (pre-vitellogenic oocytes, vitellogenic oocytes, choriogenic oocytes, and chorionated oocytes), as well as their respective FCs, we observed a tendency of higher expression of Sec16 in the more developed stages of oocytes. Furthermore, analysis of the expression of Sec16 in the FCs dissected from vitellogenic and choriogenic follicles revealed that approximately half of the expression detected in the entire follicles corresponds to the FCs, suggesting that Sec16 appears to play essential roles in the FCs later in the oogenesis process (Figure 2C).

### RNAi silencing of Sec16 leads to early lethality, disrupted oogenesis, and unviable embryos

**FIGURE 3:**
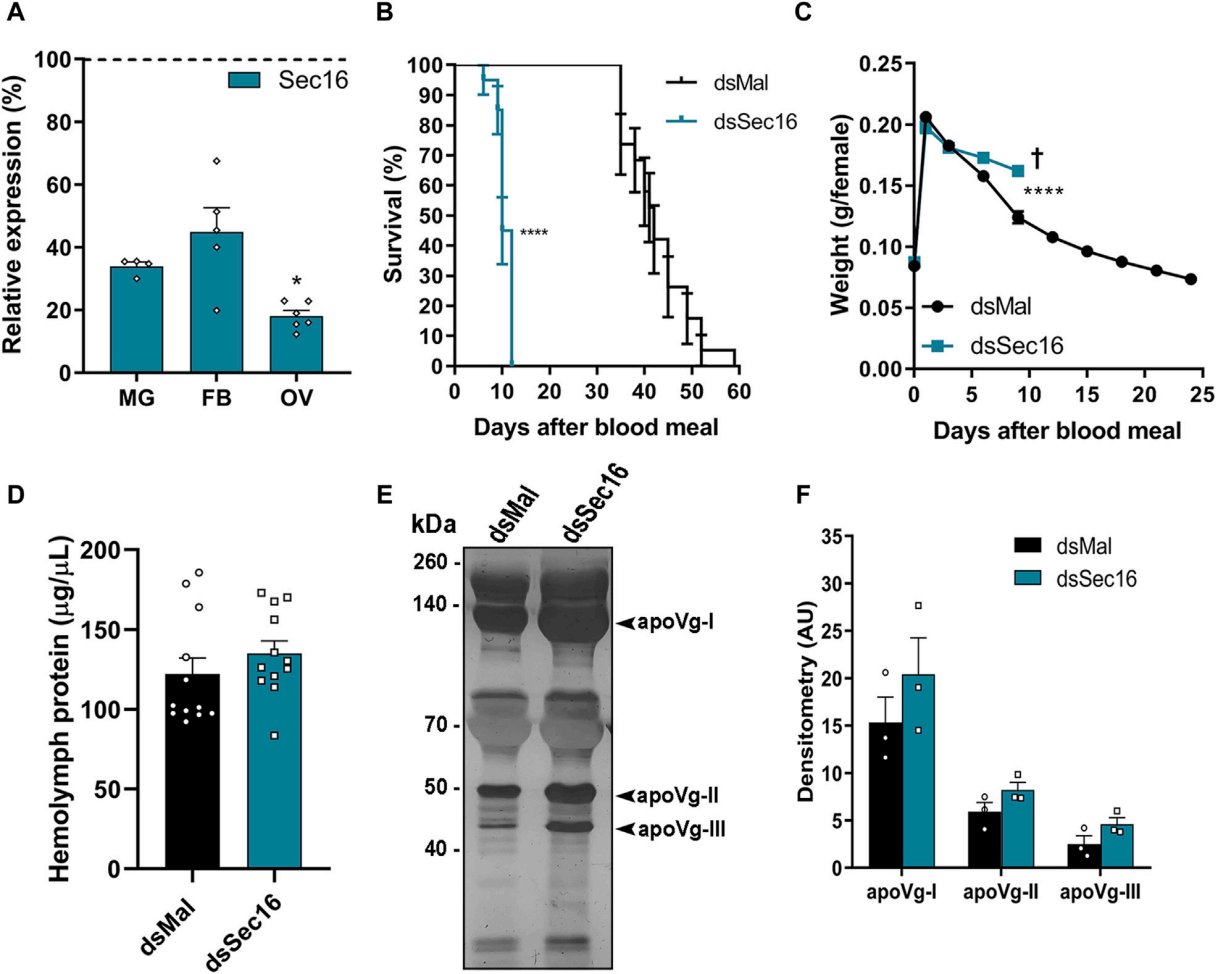
Silencing of Sec16 results in reduced lifespan, impaired weight loss and higher levels of Vg in the hemolymph. **(A)** RT-qPCR shows the Sec16 knockdown efficiencies in the different organs 7 days after the blood meal. MG: Midgut; FB: Fat body; OV: Ovary. (*n* = 4–6). *t*-test. **(B)** Survival rates of control and dsSec16-injected females (*n* = 20). Log-rank (Mantel-COX) test. **(C)** Effects of Sec16 silencing on female digestion, showing their weight during the digestion cycle (*n* = 20). Two-Way ANOVA. **(D)** Control and silenced hemolymph protein quantifications 7 days after the blood meal. (*n* = 12). *t*-test. **(E)** 10% SDS-PAGE of the hemolymphs. Arrowheads point to the vitellogenin apoproteins. (*n* = 3). **(F)** Densitometry of the Vg apoproteins apoVg-I, apoVg-II and apoVg-III shown in **(E)** (*n* = 3). *t*-test. †: Indicates the death of all individuals. AU: Arbitrary units. Graphs show mean ± SEM. *p < 0.05, ****p < 0.0001.

We synthesized specific double-stranded RNAs (dsRNAs) that were injected directly into adult females’ hemolymph 3 days before the blood feeding to further examine the function of Sec16 during oogenesis. This protocol gives the insects enough time to recover from the injection injury, so they are able to feed appropriately, and produce silenced insects since the onset of oogenesis (immediately after the blood meal). We observed that the mRNA silencing of Sec16 had an efficiency of approximately 66% in the midgut, 55% in the fat body, and 82% in the ovary on the 7^th^ day after feeding (Figure 3A). Because we discovered that mRNA silencing occurs systemically, we examined whether there had been any changes to the primary aspects of the adult female’s physiology.

We found that silencing significantly decreased insect longevity, with a median lifespan of 41 days for control females and 10 days for females silenced for Sec16 (Figure 3B). In the brief time they were alive, we observed that the female’s weight suggested a partially reduced blood digestion and/or diuresis (Figure 3C). To investigate whether or not vitellogenesis was affected, protein amounts in the hemolymph Sec16-silenced females were quantified on the 7^th^ day after the blood meal. When Sec16-silenced insects were compared to controls, no statistically significant differences were found; however, Sec16-silenced insects did show a tendency toward a minor increase, of approximately 10% (Figure 3D). When investigating the hemolymph protein profile by SDS-PAGE, we found that Sec16-silenced insects displayed a tendency towards an increase of approximately 30% in the three apoproteins of the main yolk protein precursor Vitellogenin (Vg) (Figures 3E,F). The fact that the amounts of Vg in the hemolymph of silenced females were not reduced when compared to controls indicates that the ability of their fat body to synthesize and secrete Vg into the hemolymph remained unaffected.

**FIGURE 4:**
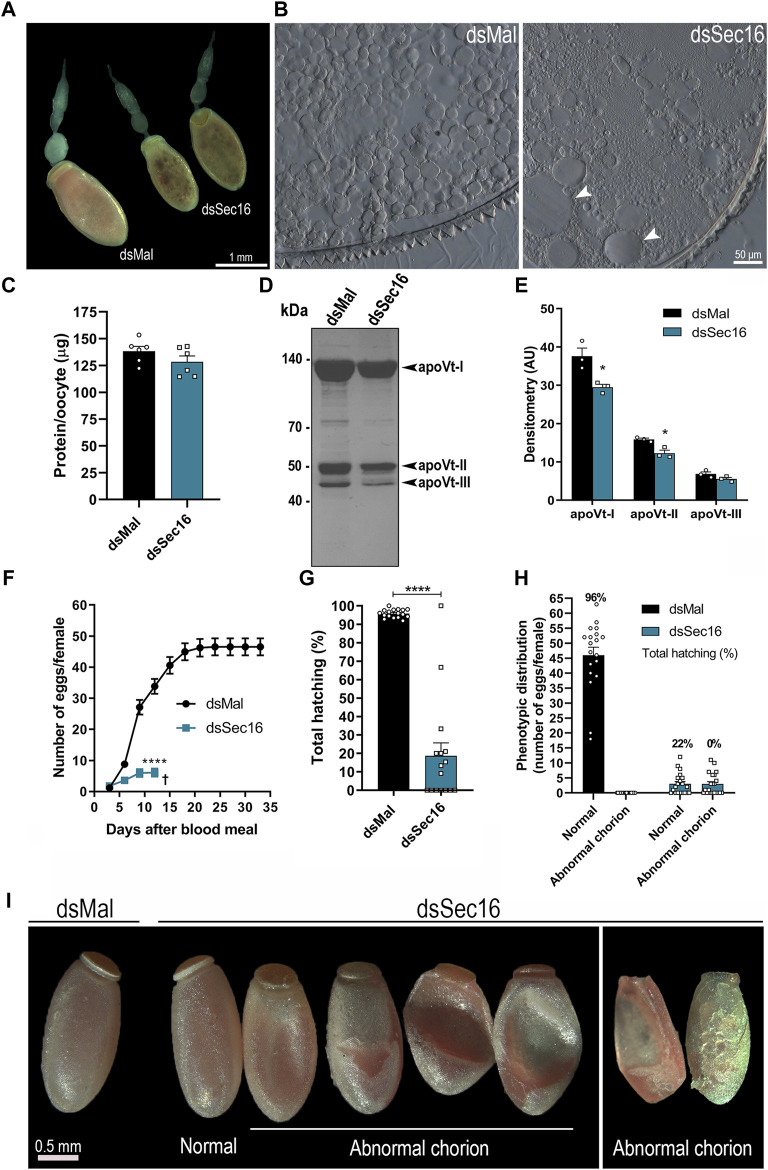
Silencing of Sec16 results in abnormal oogenesis. **(A)** Representative images of ovarioles. Scale bar: 1 mm. **(B)** Representative images of cross-sections from silenced and control choriogenic oocytes under the light microscope. Scale bar: 50 μm. **(C)** Choriogenic oocytes protein quantifications, dissected 7 days after the blood meal. (*n* = 6). *t*-test. **(D)** 10% SDS-PAGE of choriogenic oocytes. Arrowheads point to the vitellin apoproteins. (*n* = 3). **(E)** Densitometry of the Vt apoproteins apoVt-I, apoVt-II and apoVt-III shown in **(D)** (*n* = 3). *t*-test. **(F)** Oviposition of control and silenced females during the gonotrophic cycle. (*n* = 20). Two-Way ANOVA. **(G)** F1 total hatching rates after Sec16 silencing. (*n* = 20, with 884 eggs dsMal and 122 eggs dsSec16). *t*-test. **(H)** Phenotypic distribution of control and silenced eggs and their respective viabilities. (*n* = 20). **(I)** Representative images of eggs laid 24–96 h after oviposition. Scale bar: 0.5 mm. AU: Arbitrary units. Graphs show mean ± SEM. *p < 0.05, ****p < 0.0001.

When examining the dissected ovarioles from Sec16-silenced females, we found that the silencing often produced punctate smaller choriogenic oocytes (Figure 4A). Plus, their internal morphology presented abnormal biogenesis of the yolk organelles, with the presence of atypically large yolk granules dispersed throughout the cytoplasm (Figure 4B). This phenotype most likely explains the punctate morphology of the Sec16 silenced oocytes, previously reported by Bomfim and Ramos (2020) and reproduced in this work (Figure 4A). Accordingly, although the total amounts of proteins quantified in the choriogenic oocytes presented only a minor tendency towards reduction (a change that was not statistically significant) (Figures 4C), the levels of the Vitellin (Vt) apoproteins were markedly reduced in an average of 22% as measured by densitometry (Figures 4D,E). When we continued the analysis regarding the egg-laying process, we noticed that the silencing of Sec16 severely compromises the female’s oviposition rates, where a reduction of 87% (average of 6 eggs per female) was observed when compared to the oviposition of control females (average of 46 eggs per female) (Figure 4F). Altogether, these findings suggest that the higher Vg levels in the hemolymph are the result of impaired flux from the hemolymph to the oocytes, either due to the decreased number of eggs being produced (Figure 4F) and/or to their reduced uptake from the hemolymph (Figures 4D,E). Regarding hatching rates, we observed that the Sec16 silenced group had an overall 77% decreased F1 viability (Figure 4G). When we looked at each phenotype separately, we discovered that even among the eggs that appeared normal under the stereomicroscope only 22% of the embryos were viable, and those generated apparent morphologically normal 1^st^ stage nymphs. In comparison, all the eggs displaying altered chorion morphology were unviable (Figure 4H). In addition to their decreased contents of Vt, the outer surface of the chorion was evidently altered in approximately 50% of the few eggs laid by the Sec16 silenced females, including the smaller eggs, while the other half were morphologically comparable to the controls (Figure 4I).

### Knockdown of Sec16 triggers the induction of UPR-, autophagy- and UPS-related genes and reduces the expression of the main chorion proteins Rp30 and Rp45 in the FCs

**FIGURE 5:**
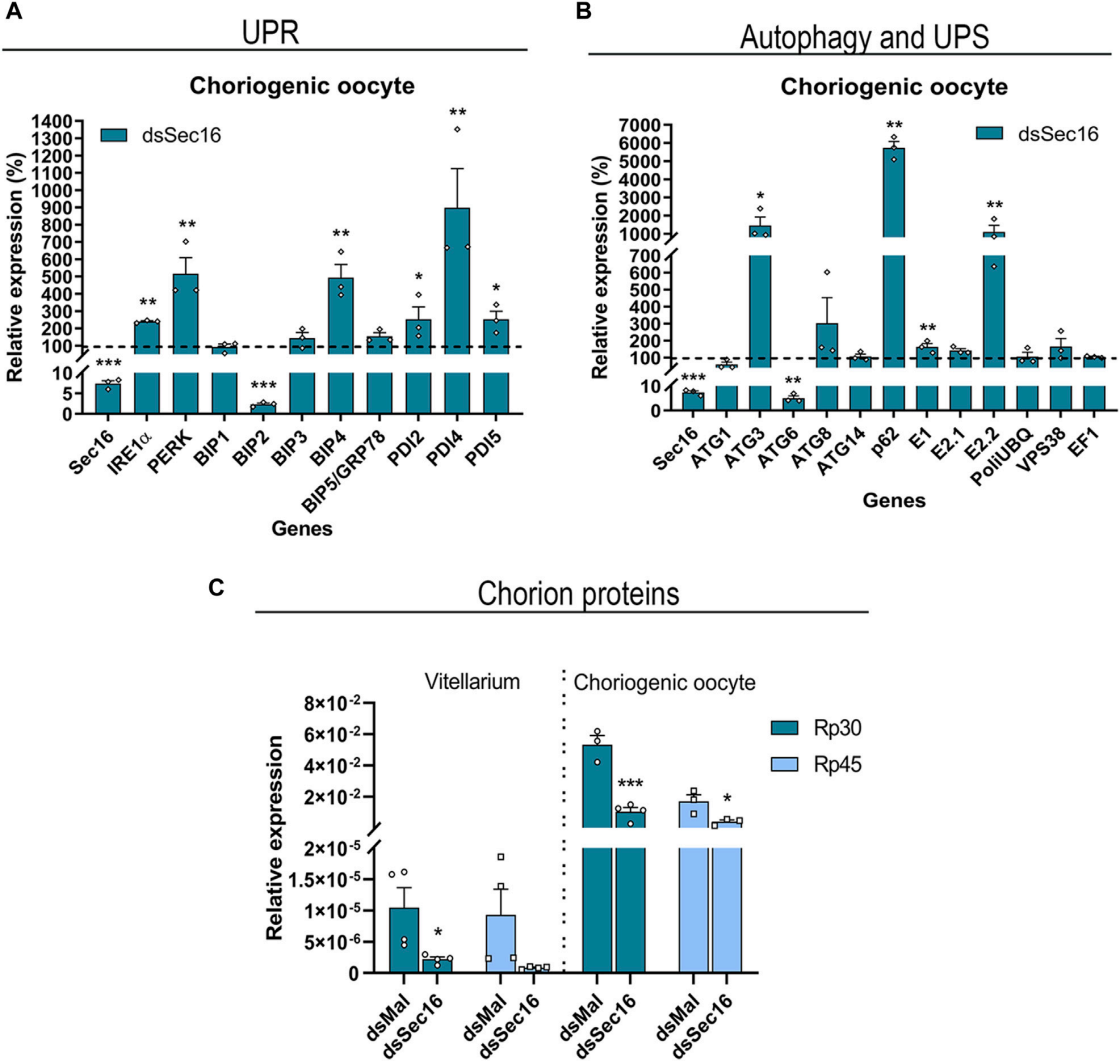
Sec16-silenced FCs upregulate UPR effectors and genes of the autophagy and UPS machinery while reducing the expression of the main chorion proteins Rp30 and Rp45. **(A,B)** Silencing of Sec16 results in the upregulation of genes related to UPR, autophagy, and UPS machinery in the FCs. **(A)** RT-qPCR showing the upregulation of the UPR sensors IRE1α and PERK and some isoforms of the chaperones BIP and PDI. (*n* = 3). *t*-test. **(B)** RT-qPCR showing the upregulation of the autophagy genes ATG3 and adaptor protein p62/SQSTM1 and the E1 and E2 enzymes of the ubiquitin-proteasome system. (*n* = 3). *t*-test. **(C)** RT-qPCR showing the downregulation of the main chorion proteins after Sec 16 silencing in the choriogenic oocyte, a follicle that contains the tissue responsible for chorion synthesis. The Vitellarium represents all the developmental stages of oogenesis except the choriogenic oocyte. (*n* = 3–4). *t*-test. Graphs show mean ± SEM. *p < 0.05, **p < 0.01, ***p < 0.001.

To further investigate the role of Sec16, we asked whether the silencing of Sec16 in the FCs would interfere with the transcription of genes related to the Unfolded Protein Response (UPR), UPS, and autophagy, all known to act in an attempt to restore cellular proteostasis (Rashid et al., 2015; Hwang and Qi, 2018). Using choriogenic oocytes, our findings demonstrated that the transcripts of the UPR sensors IRE1α and PERK, as well as the ER chaperones immunoglobulin heavy-chain-binding protein 4 (BIP4) and 3 isoforms of protein disulfide isomerase (PDI2, PDI4, and PDI5), presented significant 2–8x upregulations after silencing. Interestingly, another ER chaperone, BIP2, was downregulated (Figure 5A). Furthermore, there were significant upregulations of the selective autophagy adaptor protein Sequestosome-1 (p62/SQSTM1) of about 56x and the autophagy-related protein 3 (ATG3) of approximately 13x. There were also upregulations of the enzymes related to UPS machinery E1 and E2.2 of about 2x and 10x, respectively. Interestingly, the autophagy-related protein 6 (ATG6) showed a downregulation of 95% after silencing (Figure 5B).

Due to the above indication of disturbed cellular proteostasis, we decided to quantify the expression levels of the mRNAs encoding the two major proteins of the chorion in *R. prolixus*: Rp30 and Rp45 (Bouts et al., 2007). When compared to the levels detected in the vitellarium (tropharium, pre-vitellogenic, and vitellogenic oocytes), we discovered that both genes are 3 orders of magnitude more expressed in the choriogenic oocytes. Interestingly, both genes display similar expression levels in the vitellarium, while Rp30 is 3x more expressed than Rp45 in the choriogenic oocytes. Furthermore, the silencing of Sec16 resulted in downregulation of 79%–91% in the levels of Rp30 and Rp45 in the vitellarium and downregulation of 76%–80% in the choriogenic oocytes (Figure 5C).

### Sec16-silenced eggs present defects in chorion ultrastructure due to altered protein crosslinking and Rp30-deficiency

**FIGURE 6:**
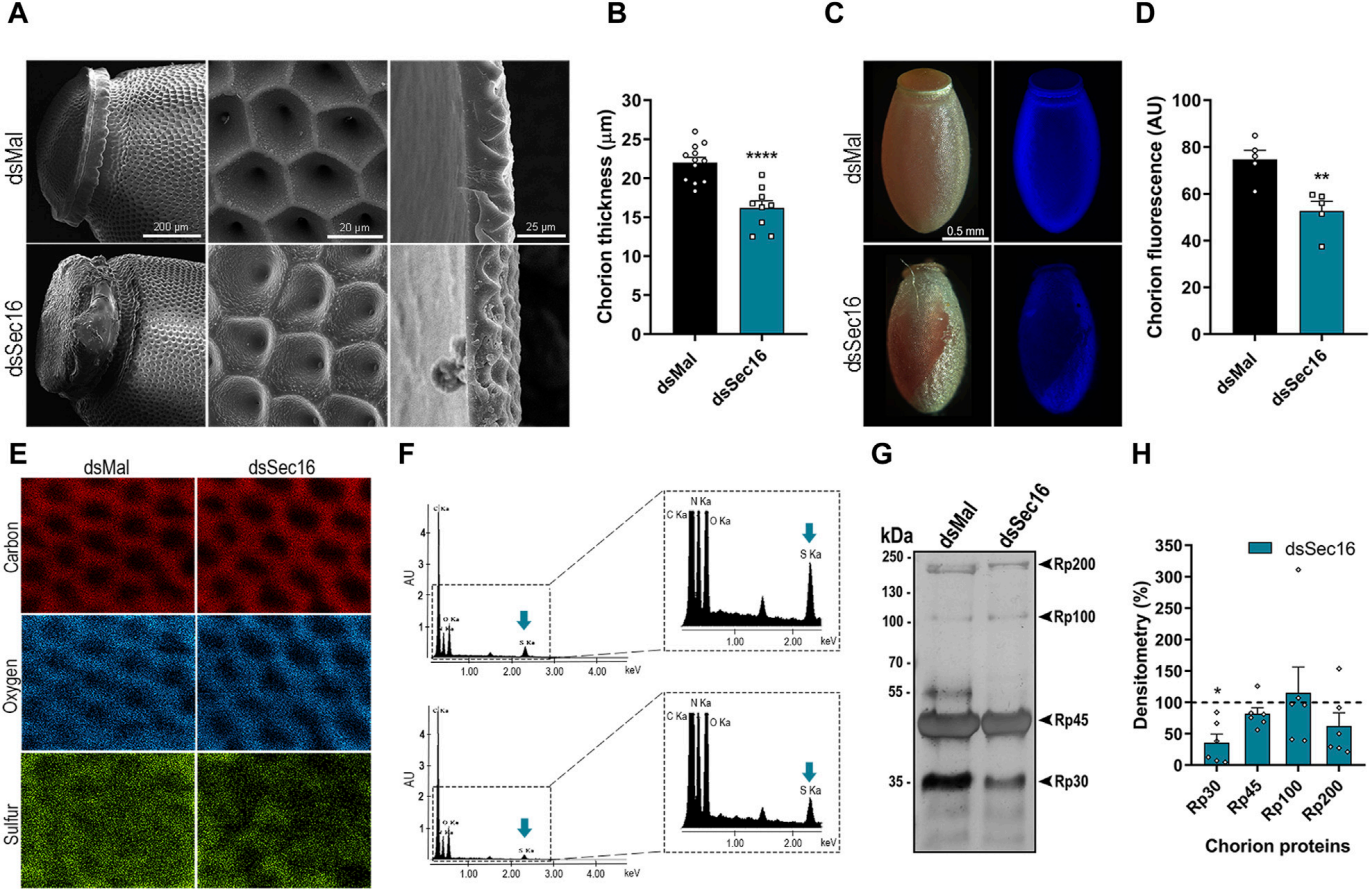
Sec16 deficiency results in abnormal chorion biogenesis. **(A)** SEM images evidencing the abnormal ultrastructure of the operculum and the exochorion surface and the reduced chorion thickness in Sec16 eggs up to 72 h after oviposition. Representative images of 4 different images. Scale bar: 200, 20, and 25 μm, respectively. **(B)** Mean thickness of the chorion of control and silenced eggs. (*n* = 12, with 3 measurements of different regions of each image). *t*-test. **(C,D)** Silencing of Sec16 decreased the tyrosine crosslinking rates in the eggs. **(C)** Representative images of the dityrosine fluorescence in eggs chorion under UV light. Representative images of 5 different control and silenced images. Scale bar: 0.5 mm. **(D)** Mean of dityrosine fluorescence quantification using ImageJ software. (*n* = 5). *t*-test. **(E,F)** Sec16 deficiency results in chorion defective elemental composition. **(E)** Effect of silencing on the distribution of elements on the exochorion surface. **(F)** Signals obtained from the spectra of the different elements. The right figure shows the spectra in an expanded form. Representative images of 5-6 different control and silenced images. Blue arrows indicate the element sulfur. **(G,H)** Sec16 silencing reduces the level of Rp30, 1 of the main chorion proteins. **(G)** 10% SDS-PAGE of chorion proteins from 0 to 72 h eggs, extracted in urea. Representative image of 3 different gels. Arrowheads indicate Rp30, Rp45, Rp100, and Rp200. **(H)** Densitometry of the protein bands indicated in G performed using ImageJ software. (*n* = 6). *t*-test. AU: Arbitrary units. Graphs show mean ± SEM. *p < 0.05, **p < 0.01, ****p < 0.0001.

After we found that the Sec16-silenced FCs presented markers of disturbed protein homeostasis and lower expression of the main chorion proteins Rp30 and Rp45, we investigated how the chorions would be affected. The eggs were prepared for scanning electron microscopy. It was possible to observe that the eggs laid by Sec16 silenced insects displayed major chorion defects in the operculum (Figure 6A, left column) as well as on the outer surface, where the distinctive pentagonal and hexagonal shapes left by the FCs impressions were replaced by irregular patterns (Figure 6A, middle column). We also looked into whether these modifications could affect the chorion’s thickness. Compared to eggs laid by control insects, which had an average thickness of about 22 μm, chorions from the eggs laid by Sec16 silenced females were thinner, measuring an average of 16 µm in thickness (Figures 6A,B).

It has already been described that the waterproofing and hardening of the eggshells of insects, including *R. prolixus*, are related to the crosslinking of proteins catalyzed by peroxidase-mediated oxidation at the expense of hydrogen peroxide. This crosslinking occurs in tyrosine residues, forming dityrosine, which emits intrinsic fluorescence under ultraviolet excitation (Margaritis, 1985; Dias et al., 2013; Heck et al., 2013). We found that the silencing of Sec16 resulted in chorions with a 30% reduction in fluorescence emission, indicating reduced levels of dityrosine crosslinking (Figures 6C,D). Additionally, we wondered if the Sec16 silencing could affect the composition and distribution of main chorion elements. Using X-ray microanalysis and elemental mapping to analyze the outer surface of the chorion, we detected significant signals of carbon, oxygen, and sulfur, the latter being a known protein marker, and found that these elements are detected over the entire surface of the chorion (Figure 6E, left panel). Sec16 silencing, however, resulted in chorions with 80.5% ± 38.8% of the levels of sulfur detected in control chorions (Figures 6E, F), indicating lower amounts of proteins within its matrix. Finally, to further study the nature of the altered chorion ultrastructure, we carried out urea-extractions of the major chorion proteins from eggs laid by control and Sec16 silenced females, as described by Bouts et al. (2007). We discovered that only Rp30 is present at lower levels in Sec16-silenced chorions (Figures 6G,H), indicating that the secretory trail of this protein is mainly affected by the Sec16 deficiency.

## Discussion

In underdeveloped nations, the high frequency of neglected vector-borne illnesses, like Chagas disease, imposes significant health and financial burdens. Recently, the necessity to rely on in-depth species-specific vector biology has been highlighted by the growing threat of pesticide resistance and climate change (which can expand endemic areas) (Shaw and Catteruccia, 2019; Wilson et al., 2020). Disrupting molecular processes required to generate viable eggs is one method of managing vector populations and the recent completion of genome sequencing projects enables functional studies that expand our understanding of reproductive processes allowing the identification of new species-specific vector control targets (Ramos and Gomes, 2022).

The initiation of the choriogenesis program, during which the FCs secrete the various layers of the chorion, is the last stage of oogenesis before fertilization. While the chorion’s primary protective function is conserved in insects, its general composition and structure have evolved in a highly species-specific manner, giving rise to a wide range of shapes and functional adaptations. The main chorion proteins in insects have been identified in models such as the silk moth *B. mori*, the fruit fly *D. melanogaster,* and the mosquito *A*. *aegypti*, and revealed to be unrelated when compared among these species (Marinotti et al., 2014; Papantonis et al., 2015). Proteins conserved in many organisms are not the best target molecules for vector control agents as they negatively affect non-target animals, including vertebrates, pollinating agricultural insects, and helpful predators. Thus, studies on the molecular biology of chorion synthesis and composition in insect vectors are invaluable in terms of generating innovative biotechnological tools as they have the potential to identify safe molecular targets that are both highly specific to a single species and essential for reproduction.

The presence of the main yolk protein precursor (Vg) in the hemolymph indicates that while the systemic silencing of Sec16 clearly has substantial effects on the general physiology of the insect (e.g., longevity), it does not influence vitellogenesis by the fat body. This data demonstrates that although the reduced weight loss observed in the silenced insects may be the result of impaired digestion, it is not restricting the absorption of nutrients necessary for the fat body to synthesize and secrete Vgs to the hemolymph. Thus, there is no reason to believe that the phenotypes seen in the process of oogenesis are the result of inadequate digestion or nutrient assimilation. In fact, the levels of Vgs in the hemolymphs of silenced females is slightly higher than the ones observed in controls. Since the net levels of yolk proteins in the hemolymph is the balance between fat body secretion and oocyte uptake, higher levels of yolk proteins in the hemolymph are usually a marker of varied situations of abnormal oogenesis such as reduced Vg uptake by the developing oocytes.

In *R. prolixus*, analyses of the ultrastructure and the transcriptome of the FCs showed a high commitment to protein synthesis, processing, and vesicle trafficking (Medeiros et al., 2011; Rios et al., 2022), indicating that choriogenesis is, indeed, a highly controlled process that follows a complex synthetic pathway. The high expression of Sec16 transcripts in the ovary of vitellogenic females compared to the midgut and fat body indicates that this gene has some participation in the oogenesis process, mainly in the choriogenesis stage due to its expressive detection in the whole choriogenic follicle and its respective FCs. After silencing, we saw that the most striking phenotypes observed were about egg laying and chorion structure. The process of chorion formation represents a remarkable model system for studying *in vivo* the biogenesis of complex extracellular matrix architectures. Nevertheless, the machinery and signaling pathways encompassing the chorion proteins synthesis, sorting and secretion in the FCs are still largely unexplored. Here, we found that the silencing of Sec16, an essential protein for the secretory pathway, indeed disturbs the ability of the FCs to properly perform the chorion deposition in *R. prolixus*.

In insects, many works have demonstrated that the disruption of different genes leads to some defect in the chorion, impairing embryo viability. Some examples of chorion-specific components are the s38 and s36 proteins from *D. melanogaster* (Velentzas et al., 2016; 2018), the EOF1 protein from *A. aegypti* (Isoe et al., 2019), and the NIChP protein from the brown leafhopper *N*. *lugens* (Lou et al., 2018). Plus, functional studies in many other genes, which do not necessarily encode a protein that makes up the chorion, such as the yellow-g, yellow-g2, and laccase2 from *A*. *albopictus* (Wu et al., 2013; Noh et al., 2020), NIFoxL2 and NIFcp3C from *N. lugens* (Ye et al., 2017) and mucin1 protein from *Spodoptera exigua* (Ahmed et al., 2021), also resulted in chorion malformations. In *R. prolixus*, ultrastructural changes in the chorion have already been reported by the depletion of genes related to different pathways, such as ATG1 and ATG3 (Bomfim and Ramos, 2020; Santos and Ramos, 2021), the E1-activating and E2-conjugating ubiquitin enzymes as well as the alpha6 subunit of the 20S proteasome (Pereira et al., 2022; Faria-Reis et al., 2023), the UPR sensors IRE1α and PERK (Rios et al., 2022), Bicaudal C, a conserved embryo development regulator (Pascual et al., 2021), the acetyl-CoA carboxylase enzyme and Brummer lipase, related to lipid metabolism (Moraes et al., 2022; Arêdes et al., 2024) and Duox, a NADPH oxidase (Dias et al., 2013). These findings, when combined, indicate that chorion synthesis in insects is a susceptible process, as the silencing of genes linked to various pathways produced similar phenotypes.

The fact that we observed an altered chorion’s ultrastructure accompanied by 1) a decrease in thickness, 2) decreased dityrosine crosslinking, 3) reduced sulfur signals, and 4) lower amounts of the protein Rp30, indicates that the protein composition of the chorions from silenced insects is compromised, either due to non-synthesis or lack of delivery by the FCs to the chorion. The protein homeostasis of a cell is regulated by the balance between synthesis and degradation (Brehm and Krüger, 2015). FCs are highly secretory, so an efficient synthesis and secretion machinery is required, as previously shown (Medeiros et al., 2011; Rios et al., 2022). On the other hand, there is accumulating evidence that protein synthetic activity and the UPR are constantly and finely adjusted, so that numerous feedback mechanisms ensure efficient adaptation to fluctuations in protein synthesis and folding requirements, including the activation of degradative pathways such as autophagy. Thus, the coordination of the UPR and degradative pathways, such as autophagy and the UPS, is also likely vital to maintain proteostasis in the FCs and allow proper chorion biogenesis (Bomfim and Ramos, 2020; Rios et al., 2022; Santos and Ramos, 2021; Pereira et al., 2022).

Based on the results of upregulation in the expression of UPR, autophagy and UPS related genes, as well as the downregulation of the main chorion proteins Rp30 and Rp45, we hypothesized that the silencing of Sec16 led to a retention of cargo that should be exported from the ER via COPII vesicles, causing the activation of the UPR and degradative pathways (Kozutsumi et al., 1988; Kroeger et al., 2012). Interestingly, while the upregulation of many UPR, autophagy, and UPS genes was observed, the oocytes of Sec16 silenced insects also showed significant downregulation of the isoforms BIP2 and ATG6, indicating a complex cellular response. ATG6 is a highly multifunctional protein that has been primarily identified as a gene associated to autophagy, but it also plays functions in endocytosis, aging, immunity, and cell death (He and Levine, 2010). Similarly, several interaction partners such as Hsp40 co-chaperones, nucleotide exchange factors, and signal transducers are necessary for the many isoforms of BIPs to function (Dudek et al., 2009). Therefore, it's plausible that the downregulation of those particular genes has something to do with their functional complexity, which includes a large range of interaction partners and multiple activities within the cell.

It is important to emphasize that phenotype interpretation may require careful consideration due to the likelihood of indirect effects resulting from the systemic gene silencing. On one hand, the findings of significantly larger yolk protein concentrations in the hemolymph of vitellogenic silenced females suggest that vitellogenesis was not seriously hindered, even though the midgut and fat body were both silenced for Sec16. However, since we did not investigate gene silencing or the morphology of the Malpighian tubules from silenced insects, it is not possible to rule out the possibility that the observed reduced weight loss after blood meal is the result of impaired postprandial diuresis (Orchard et al., 2021) rather than poor midgut digestion, and that poor diuresis caused indirect deleterious effects on oogenesis. In addition, previous findings in the literature describe that silencing components of the COPII machinery can disturb oogenesis in insects, such as the beetle *Colaphellus bowringi* (Tian et al., 2022).

**FIGURE 7:**
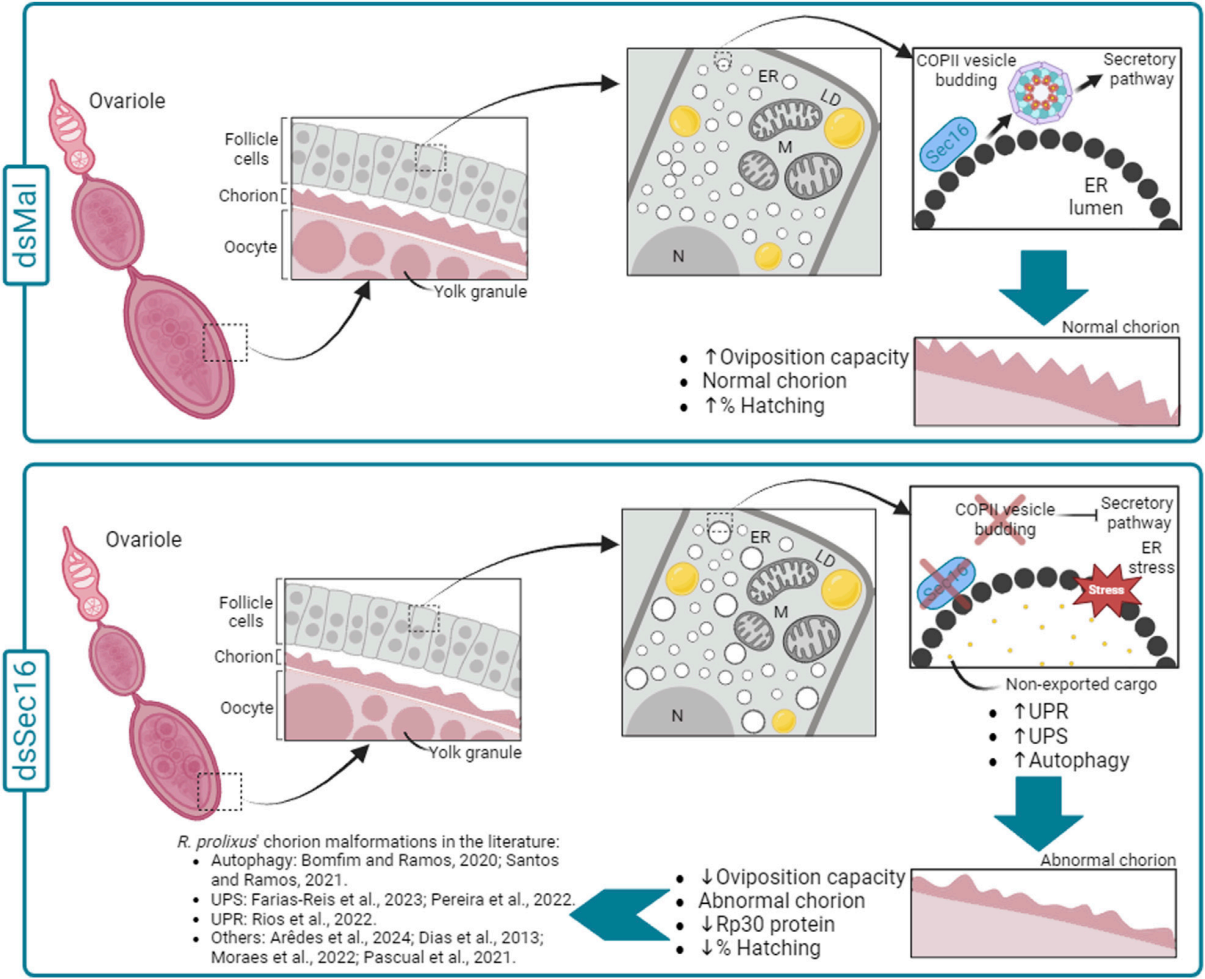
Schematic diagram summarizing the effects of silencing Sec16 in the chorion formation in *R. prolixus*. In the control condition (dsMal), during the choriogenesis process, the FCs act in the synthesis and secretion of the chorion components. Within the FCs, chorion proteins are directed from the ER to the Golgi apparatus via COPII vesicles, proceeding through the entire secretory pathway. This pathway allows the correct export of the chorion components to the extracellular space and their deposition over the oocyte cell membrane, enabling the production of eggs with a rigid eggshell capable of protecting the developing embryo. On the other hand, in the silenced condition (dsSec16), our data suggest that Sec16 silencing results disturbed proteostasis (ER stress) in the FCs under due to the hindered exportation of the cargo through COPII vesicles. This disruption is evidenced by the upregulation of genes involved in cellular proteostasis pathways (UPR, UPS and autophagy) and the expansion of ER lamellae compared to the control condition, consistent with previous findings by Bomfim and Ramos, 2020. As a result, the faulty functioning of the FCs leads to the production of malformed chorions, with reduced thickness and lower amounts of the Rp30 protein, culminating in reduced embryonic viability rates. In addition, the silencing of several other genes has been previously reported to culminate in chorion malformations, indicating that the coordination of choriogenesis by the FCs is a complex and sensitive process in *R. prolixus*. ER: Endoplasmic reticulum; LD: Lipid droplets; M: Mitochondria; N: Nucleus.

In conclusion, the findings from this study highlight the multifaceted role of Sec16 in *R. prolixus* oogenesis and reproduction. Sec16 is essential for ensuring proper protein synthesis and secretion during oocyte development, influencing eggshell formation. Its depletion triggers a complex cellular response, including ER stress, activation of protein degradation pathways, and alterations in chorion protein composition and crosslinking. This research contributes to our understanding of the molecular mechanisms underlying reproductive processes in *R. prolixus* and may have broader implications for our understanding of oogenesis in other insects. Figure 7 displays a schematic model summarizing our discoveries.

## Data Availability

The original contributions presented in the study are publicly available. This data can be found here: GenBanK accession number: BankIt2819959 Rp-sec16 PP683476.

## Supplementary material

SUPPLEMENTARY FIGURE S1 18s as a stable reference gene. 18s Cts were obtained from different organs after the silencing of Sec16. Total RNA was extracted from the ovary, midgut, and fat body. All samples were dissected 7 days after the blood meal. Graphs show mean ± SEM (*n* = 4–7).

SUPPLEMENTARY FIGURE S2 Assembled Sec16 full ORF. **(A)** Schematic diagram showing the conserved domain Sec16_C (pfam12931) and primers designed to test the complete newly assembled sequence of Sec16. The graphic was generated using the DOG 2.0 software (https://dog.biocuckoo.org). The conserved domain was identified using Pfam 35.0 (https://pfam.xfam.org/). **(B)** Table showing the size of the amplicons expected for each pair of primers. **(C)** 2% agarose gel showing the results of the PCR products using the nine pairs of primers listed above. **(D)** Percentages of similarity and identity between Sec16 sequences from different species. R. prolixus (RpSec16); *D. melanogaster* (DmSec16); *A. aegypti* (AaSec16); *C. lectularius* (ClSec16); *Mus musculus* (MmSec16); *Homo sapiens* (HsSec16).
