# Supplementary material for: Knockdown of Sec16 causes early lethality and defective deposition of the protein Rp30 in the eggshell of the vector Rhodnius prolixus
Source: Front Cell Dev Biol. 2024 Apr 22;12:1332894. doi: 10.3389/fcell.2024.1332894 (PMC11070790; doi:10.3389/fcell.2024.1332894)
Supplement: Supplementary file 1 [file Table1.DOCX]

**Table S1: Genes and primers list.** All sequences of primers used in qPCR experiments and dsRNA synthesis. All primers were synthesized by Exxtend. The T7 adapter sequence is underlined.

| Gene | Vector Base | Primer sequence (5’-3’) | Amplicon (bp) | Reference |
| --- | --- | --- | --- | --- |
| Sec16_1  (qPCR) | RPRC002699 | FOR: TTCACCAAGAGGAGCAACAG  REV: GAGTAGGAATTTGATGTTGG | 151 | [1] |
| Sec16_1  (dsRNA) | RPRC002699 | FOR: GGCCGCGGGGCAATTGATCATGGTGCTTGGG  REV: CCCGGGGCGCTAATCCAGCATCATAGTCCTC | 768 | [1] |
| Sec16_2  (qPCR) | RPRC002702 | FOR: TTATTCCCAACTCCTGCACA  REV: TCTGCTTGATTTCCGTTGTTC | 151 | [1] |
| Sec16_2  (dsRNA) | RPRC002702 | FOR: GGCCGCGGAGCAGAATGCAGGATGGTTTGG  REV: CCCGGGGCCCATCAATAGGGCGAACTGTAC | 355 | [1] |
| 18s | RPRC017412 | FOR: TCGGCCAACAAAAGTACACA  REV: TGTCGGTGTAACTGGCATGT | 105 | [2] |
| IRE1α | RPRC008861 | FOR: GGGGAACCAATAAGAAGACAAAG  REV: CATCCTGAAGGAAGGTTAATGTG | 208 | [3] |
| PERK | RPRC004775 | FOR: CGCCTGGTATCAGCACTTCTTCG  REV: CCACAGCTCTGACGATTTGATTG | 171 | [3] |
| BIP1 | RPRC009759 | FOR: GAGCGCAACGCTAGAATACC  REV: TGGCATCCAGATCGAATGTA | 205 | [3] |
| BIP2 | RPRC006365 | FOR: GGTATCCCCCAGATTGAGGT  REV: TGCGTTTCTAGCATTGATCG | 216 | [3] |
| BIP3 | RPRC010252 | FOR: AGGTGGTGGAACCTTTGATG  REV: GATGATAATGCTCGCTTGGC | 228 | [3] |
| BIP4 | RPRC004310 | FOR: CTTATGGAGCTGCTGTGCAA  REV: CTCCGGGTTGGTTATCTGAA | 205 | [3] |
| BIP5  (GRP78) | RPRC013386 | FOR: CTAACACCGGCAACACCTTT  REV: ACTCTCCGCTGTTTCCTTCA | 213 | [3] |
| PDI2 | RPRC004119 | FOR: TCACTCTTGCCAAGGTCGAT  REV: TTCGCACTTGGACCAACTTG | 181 | [3] |
| PDI4 | RPRC010401 | FOR: TTTCACTGGAGGCCTAGACG  REV: GGATAGAGTGCAGCCGTTTG | 208 | [3] |
| PDI5 | RPRC002610 | FOR: GGAATCAATCTGCACCGAGT  REV: CGCACAACAGATGGAGTAGC | 179 | [3] |
| ATG1 | RPRC009624 | FOR: GTACTGGCGTTGAGTGAATGTG  REV: CATTGCTTACTGTAGGCGATGG | 166 | [1] |
| ATG3 | RPRC008742 | FOR: CCAGAAGAATTTGTTGCTGCG  REV: CGATTGATAGACCCCGACGATCC | 203 | [4] |
| ATG6 | RPRC006439 | FOR: CCGCTCCTGTAGACTGGTC  REV: GCCACCATCGCAGCATCAAATTTTG | 226 | [5] |
| ATG8 | RPRC014434 | FOR: GAACAATGTAATCCCACCGACAAG  REV: CCATAGACATTTTCATCACTATACGC | 108 | [6] |
| ATG14 | RPRC001958 | FOR: TTGGGTTGCAGCGAACAAAG  REV: TTCAGTCTGGCTACGCGTTT | 219 | [7] |
| p62 | Non annotated | FOR: AATGACGTTTTGAAGGCGGG  REV: TTGCTCGATGTTGCCGTTTT | 225 | [8] |
| E1 | RPRC003935 | FOR: GCTCCAAAACTCAAGTACTATGAG  REV: ACAAGTGCTCTAACATGCGGTTCA | 263 | [9] |
| E2.1 | RPRC007710 | FOR: AGAAGCGGCTGAAGTTTTGC  REV: GAACGCGGACAATCGCAGTA | 156 | [9] |
| E2.2 | RPRC006315 | FOR: TTACCCATCCAGTCCTCCAAAA  REV: CTCTTTTCATACTCAAGCCTGT | 240 | [9] |
| PoliUBQ | RPRC012247 | FOR: AAGGAATTCCACACCAACA  REV: GAGGGCTCAACTTCAAGAGT | 188 | [9] |
| VPS38 | RPRC001388 | FOR: TTCGCCCACAGCCTAAAGAG  REV: ATCGGGAAGATGAACCCCTC | 323 | [7] |
| EF1 | RPRC007684 | FOR: GATTCCACTGAACCGCCTTA  REV: GCCGGGTTATATCCGATTTT | 92 | [7] |
| Rp30 | Non annotated | FOR: GTAGACCTGTTGTTGACTCC  REV: CTGGCGTAATGCTGAGTGAC | 102 | Not published |
| Rp45 | Non annotated | FOR: GCTCCTGCTGTCTACCAT  REV: CTCCGACATAGCCAGCAG | 109 | [10] |

1. Bomfim L, Ramos I. Deficiency of ULK1/ATG1 in the follicle cells disturbs ER homeostasis and causes defective chorion deposition in the vector Rhodnius prolixus. FASEB J Off Publ Fed Am Soc Exp Biol. 2020 Oct;34(10):13561–72.
2. Majerowicz D, Alves-Bezerra M, Logullo R, Fonseca-De-Souza AL, Meyer-Fernandes JR, Braz GRC, et al. Looking for reference genes for real-time quantitative PCR experiments in Rhodnius prolixus (Hemiptera: Reduviidae). Insect Mol Biol. 2011;20: 713–722.
3. Rios T, Bomfim L, Ramos I. The transition from vitellogenesis to choriogenesis triggers the downregulation of the UPR sensors IRE1 and PERK and alterations in the ER architecture in the follicle cells of the vector Rhodnius prolixus. Cell Tissue Res. 2022;387(1):63–74.
4. Santos, A., & Ramos, I. (2021). ATG3 Is Important for the Chorion Ultrastructure During Oogenesis in the Insect Vector Rhodnius prolixus. Frontiers in Physiology, 12.
5. Vieira PH, Bomfim L, Atella GC, Masuda H, Ramos I. Silencing of RpATG6 impaired the yolk accumulation and the biogenesis of the yolk organelles in the insect vector R. prolixus. PLoS Negl Trop Dis. 2018;12(5):1–19.
6. Pereira J, Diogo C, Fonseca A, Bomfim L, Cardoso P, Santos A, et al. Silencing of RpATG8 impairs the biogenesis of maternal autophagosomes in vitellogenic oocytes, but does not interrupt follicular atresia in the insect vector Rhodnius prolixus. PLoS Negl Trop Dis. 2020;14(1):e0008012.
7. Vieira, P. H., Benjamim, C. F., Atella, G., & Ramos, I. (2021). VPS38/UVRAG and ATG14, the variant regulatory subunits of the ATG6/Beclin1-PI3K complexes, are crucial for the biogenesis of the yolk organelles and are transcriptionally regulated in the oocytes of the vector Rhodnius prolixus. *PLoS Neglected Tropical Diseases*.
8. Faria-Reis A, Santos-Araújo S, Pereira J, Rios T, Majerowicz D, Gondim KC, et al. (2023) Silencing of the 20S proteasomal subunit-α6 triggers full oogenesis arrest and increased mRNA levels of the selective autophagy adaptor protein p62/SQSTM1 in the ovary of the vector Rhodnius prolixus. PLoS Negl Trop Dis 17(6): e0011380.
9. Pereira J, Dias R, Ramos I. Knockdown of E1- and E2-ubiquitin enzymes triggers defective chorion biogenesis and modulation of autophagy-related genes in the follicle cells of the vector Rhodnius prolixus. J Cell Physiol. 2022;1:12.
10. Medeiros, M. N., Logullo, R., Ramos, I. B., Sorgine, M. H. F., Paiva-Silva, G. O., Mesquita, R. D., Machado, E. A., Coutinho, M. A., Masuda, H., Capurro, M. L., Ribeiro, J. M. C., Cardoso Braz, G. R., & Oliveira, P. L. (2011). Transcriptome and gene expression profile of ovarian follicle tissue of the triatomine bug Rhodnius prolixus. *Insect Biochemistry and Molecular Biology*, *41*(10), 823–831.
