# Supplementary figures and images for: Knockdown of Sec16 causes early lethality and defective deposition of the protein Rp30 in the eggshell of the vector Rhodnius prolixus
Source: Front Cell Dev Biol. 2024 Apr 22;12:1332894. doi: 10.3389/fcell.2024.1332894 (PMC11070790; doi:10.3389/fcell.2024.1332894)

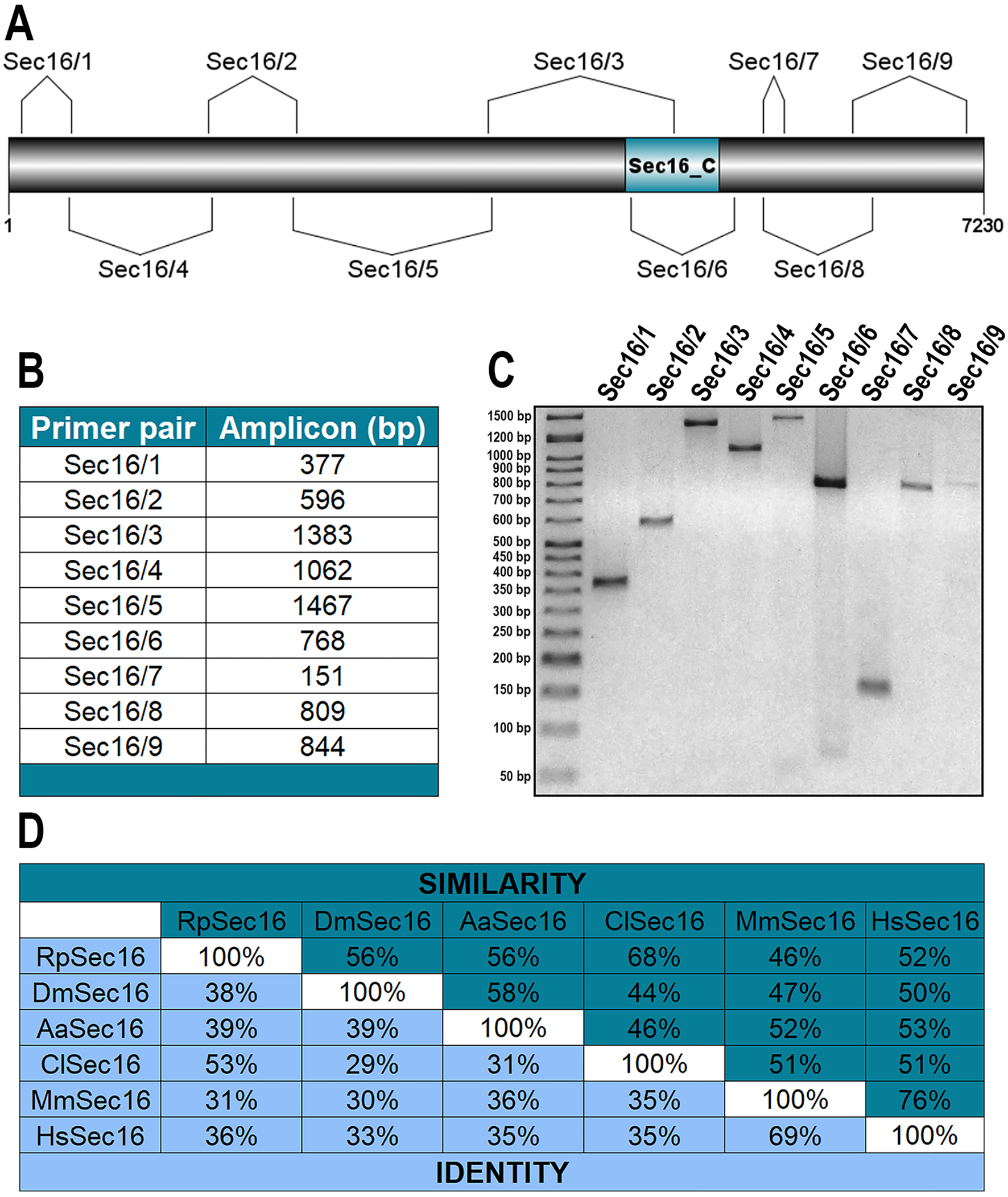

Supplement: Supplementary file 2 [file Image2.TIF]

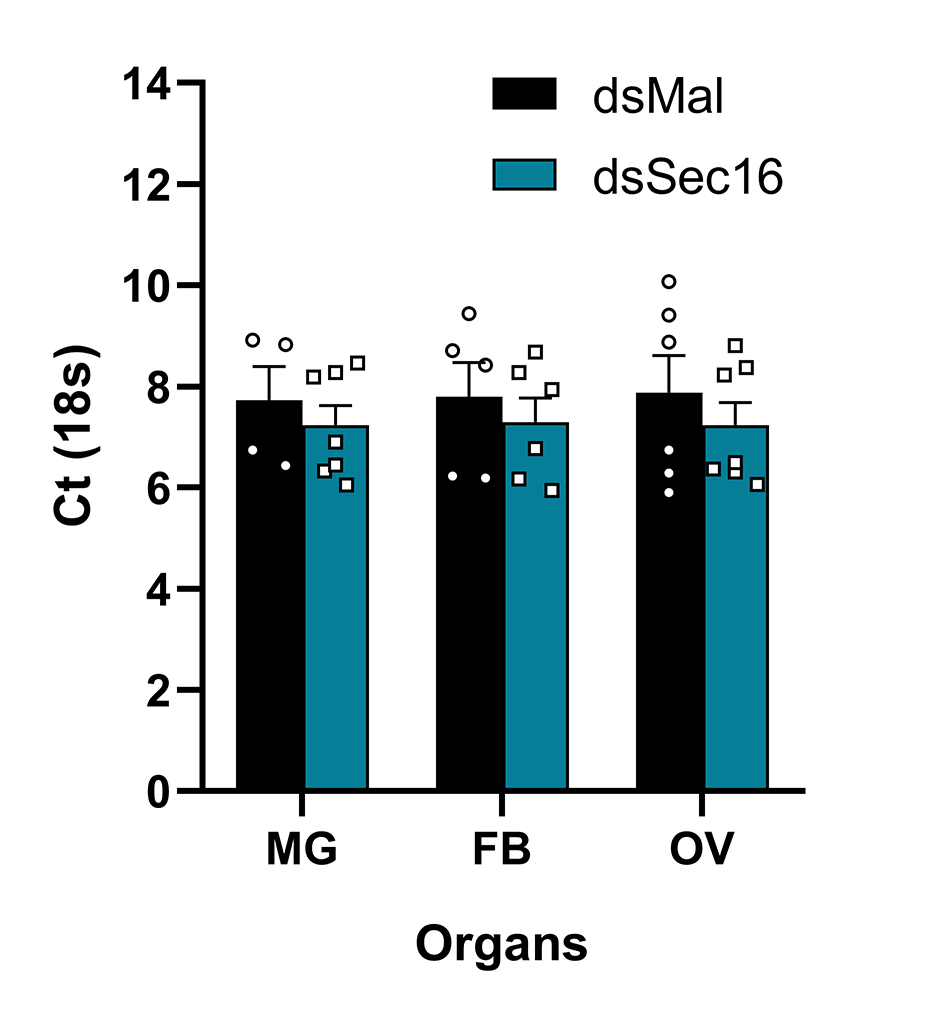

Supplement: Supplementary file 3 [file Image1.TIF]
