# Supplementary material for: Knockdown of Sec16 causes early lethality and defective deposition of the protein Rp30 in the eggshell of the vector Rhodnius prolixus
Source: Front Cell Dev Biol. 2024 Apr 22;12:1332894. doi: 10.3389/fcell.2024.1332894 (PMC11070790; doi:10.3389/fcell.2024.1332894)
Supplement: Supplementary file 4 [file DataSheet1.PDF]

***Rhodnius prolixus* Sec16 sequence**

- **Nucleotide sequence:**

Legend:

Underline: The protein open reading frame.

RPRC002689

RPRC002699

RPRC002702

>*Rhodnius prolixus* Sec16

AGCTGAATTTTGACTGCCACTTCGCTGTTGTTAACCATATAGACAGTATCCGAGAATATGAATC

ATAGCGAGCAAGACCCTTGGACTTGGGGCTATGAGGATAGTACTGTTGAAGATATCCAACATAA

TGAATGGAATGCTTGGAATACCTGTACTCCATTAACATCCGATAATAAGAAAAATAAGGATAAC

TCTAATAAGGTAAAACATCCACCAAATCAAGGATCTGAATACTTCGATACATTTAATTTGAGAA

ATACTTCTCAAAATGTCCAACATAACTGGACTTCTGGGGATGCAATGCAGCCCAACTATGCTAA

TTTTGTATCCCATTCTAACACTTCAAATTATTTTAATAATAATTGCCAGACTGACGTTGAAAAT

AAAGAAGTGGCGCCACCAGATAATCAATACAATAGGAATTCTCATCAGCCGCCATTGCCGGAGG

ATTCTAAGCGTGATCGTCACATTCAAGAACAGATGACAGTTCCTACATTTAATCAAAAAAAGAC

AACGCCCATTGCAGCTCCCTTATTAGGGCCTTCATTTGGTTCTAAACAATTTGATCCCCAGGAC

AATATGTTCCAGCAGCCAGTTAAACATCCGAATTCTATGTCTAGTGCCGTAGCAAACGTTTCCT

ACAACCCCGTAGGTCAACATTACAGTACTACAACTCCCTATTCTAAAGAGCAATCGTTTTATTT

CCCATCTTCTTTATCACATGAAGGTGCAACAGGATTTCAAGCCCATTCAAATAGTTTGAGTGAT

TTACCTTCTCGACAGCAGAATGAAAGTAACAACTCCCTGAGCGGTCCACAATCACTACCTCCAT

TTCATTCCTCCTCAGTTTCTTCGCTGAGTAATAATTCTACTGATCACATTATACAGCATTCAAG

TAGTACGTCTTCAACAATTTCCGATGCCTTTCATACCGAAAAATCTCGTCATGCTCATGCTGTA

TTACCTTTAAATGATAACTCTTCGCAAAATAAGCATCAACAAATGCGCGATTTATCCCAGACAG

GGAAAATGAAATCTCATTTTTCTCAACAAAATGTTTCTAGTAAGGAGCAAGCAAGTGTTTTCAG

TTCTGCCGTAGCGGATAAAGATGTGGCTTCAAATCGACATGAATTTTCGACTCATTCATCCAGC

TTAGTTGATTTAGCTGAAGACGTTAGTAACTTTTATCACCCATCTAAACAAGAAATTAAAGAAG

GTGATCAGACCCAACTGCAGCAACAGTTAGATTTCCATGGATCTCATAAGTCTGAGCCACCTCA

GGTTTCTAATCAAATGTCTGCTTTTAAGCCATCCTTAGCTCGACCTCCCCTTATTCATCAAATG

CCAGTTTCAGATTCTCAACAAATATCTGTTTCACAGCATCTTCCCGTTCTTCAGCAACCTCCTT

TTCCTCAGTCCTCGGCAGATCCTCAGCACCGTTCCCTTTTCCATCATGTACCTGCTTCTCAACT

CCACCACGTTCCTCAACAACACACTTCGCAGCAGCTTGCTGTTAATCAGAGTTTACCAACTGTA

CAACAGTCTCCTATTTCTCAACATTTACCAGTTTCCCAGGGTCCAACCATTGATCAGCATACTC

CTGTTTCCCAAAATCTCCCAGTTTCTCAGGACCAAAACATTGTTCATCACCCTACAATTTCTCA
ACATCTCCCTGTTTCTCAGGACCAAAACATTGTTCATCACCCTTCAATTTCTCAACATCTCCCA
GTTTCTCTGGACCAAAACATTGTTCATCACCCTTCAATTTCTCAACATCTCCCAGTTTCTCTGG
ACCAAAACATTGTTCATCACCCTACAATTTCTCAACATCTCCCTGTTTCTCAGGACCAAAACAT
TGTTCATCACCCTTCAATTTCTCAACATCTCCCAGTTTCTCAGGATCAAAACATTGTTCATTAC
CCTCCAATTTCTCAACATTTACCTCTTGCTCAGCACCAATCAGTTGCGCAGCAACCATCTAATT
CTCTTTCTCATCATCTGTTGGTTTCTCAGCAACCTCTAATAACTGCGGATGCCACGATTCCTCA
TCAATCATTAAGTTCTCTACAAAATTCAGTTTCTCAACACGTAATGCCTCAGAGCCACCCACAT
TCTCAGCAAACTCCAGTATCTCACTACCTTATGGCTTCTCAACAACCTTCTGGGCCTCAACAAC
CCTCTACGTCTTCTCTACCTGTCCCAACTTCTCAGCAATCTCAGATTTCTCAAGAATCTCCAGA
TTTCCTGTCATCCCCATCTGTTCAACAAACTCAGGTTTCTCAACAACTTACAATTTCTCAACAG
TCCCAAGATTCTAATAATGTTCAAGAAAATCCTGATACTAAGAATTTTTTTCAAATCTTTGTC
AAAGGGATGACCATCCACAAGAAATTATTATGCCTGATTCTCAAAAAATTGAGACCAATAATTT
AAAACTCTTACAGAATGTTTCTGTTAGAAATTTTAATTCTATATCTGTCGATGATAGCCAGATA
AGCCAAGTTGATTTACTGCCGAACAGTCAACAAAGAGATAAAGATACAACACTTATTACTCAAA
ATAGTAACAATTCATTAACTGAATTAAGAAAAGAAGCCACAGAAACTGTGCAACTTGATAATGA
TACAAGTAATTTTAATGCAAACACCAGTAGGAACCAGATGTCTTTTAACTTTCAAAATGTACAT
CCGGTAGCTTCTAGCAGTCCCGTTCCTTCACATAACATTCCAGACAATCAAGAAAGACTACCCG
AAAACCATGAGTTGCCCGATAATACTGAAAATTTACATCTTGATATGAAACAGTTGACACTGAA
GAGAGAGGTAGAAAGTGATTCTCAAATTCACTGCCAGGTAGACCGTAATCAGTATCTAGAAACA
GGCCATCTTTCTGGTTCTAAAGATCACATTTTATCAGATAATTCGCCAAACTTTGAAGCAGTCG
ATGAAGGCGAAGCACCTCCACCAGGACTTCATCGATTAGTGACCGGTCAAGGCTGTGAAACTAG
AGGTCCTAAGACAATTAGTGATCATCAATTGCTTATGGTTGGTGCCGATTCCACAATAGTTGAC
GACCATAGACAAAGTTCTTTATCATCTTCAACTGTTGGTTCGGTTTCAGATAATAGAAATGCTC
AAAATGGGCCACCGGTAGGGGAAAGTGAAGTACACACTCGAACGGGAAGATTAGTACAGGGACA
AAGTATAGGAGATGACACAGACGGTGTAAGAGAGGTACCTGGAGAAGCTACACCATTTCAAAAT
AGAATTGTTCTGGGTCAAATGGGAAGAGGTATAACCCCTCCACTCGTGCAAGACAACAGTACAA
CTAGAGGAGATAATTCGAGAGAAAGAGAAATCGTTGGACGTATGGTTTTAGGTGAACGTTATGA
TGACAGAGACCTTACACAGAACGTCAATTCTCAGGACGATTTTAGGCAATCCCATAGGAGTTTA
AGAAGAAGCAGAAGAGGAGAGAGTAGTTATGAAGATGAAGAGCATGATTATCCTAGCGACAGAG
ATCGGCGTGTTGATGAATATAAAAGAAGAGGAGATGACAGAGTACGACATAGGAATCGACGAGA
ACACAGATCTCCAGAGTATAGAAGCGATGAGGAATTTGAAGATCGACGAGGTTTTAGTAGAATG
GGAAGAAGTGAGAGGAGGCCTCGTGAGTTTTCACCTGACATGTCGTATTACAATAACAGTTATT
ACAGAGACGATCATAGAAGACATAGAAGATATGACCATTACAGAGATGATTATGATGATTATTA
TTATCGAGAAAATAGATCGCGACCATCTAGTAGAACAGGATCTGACTATAGGCGTATGAATATG
GACTATTATTCTGGAAGGGTCAACGCAAGGCCTATGTTTTACTCAGATTTAGTAGGAGCAATTC
CTTACAATCCTCGGGCGCCAGAAGAATATTTCGAAGCTATGAGAAGACTTGATCCTATGGATA

TGCCGCATGGTACAACCAATACATGAATAGTAGATATAATGTTCAACAGTCCCAATCAAACTAT
AACAACGACAGAGCAAGCGTTCATTCCGGCCAGAGCTCTACTAATAATCAACGTCCAAATGTTG
AAACTCCACAAGCTGGAGTGGGGGAAGAAATTGGCAGGGATGAGGAAGAATTTGTACCTGCTAC
TGCTCATATTAAAGGAATAATTGACAATTATGGTCGTTTGGTTGTAATTGACCCTAATTATTCA
ATGGATAGTAAAAGACGTAACATCAATATTTATCAGATATCTAAATTGCAATCTGATCCTGATC
TTGATGAATTCTTAGAAAGCCCTGGGCCTTTTATACCAGGGGTAACTCATAGAAACACAGTTTT
GCAATACCTCAAAAGGATTTCTGAAAGAAGCACTAAAAGTTCAGAAAAATTACTTTACGATTTA
GTTCACTTGTCTGTAAAAAGTAATGGGGAACTTAATGGACTTGATGTGGCCGATTTGCTGATGG
AGAGTTACAAAAAATCTCAGTCTACAGAAGCCCCTAATTTGTCTGAACCATTAGTCGAACAAAT
TTCTTCAACAGATGCCCTAACGAAATTCAGAGAATTATTGCAACAAGGAAATAAAACAGAAGCT
TTAGAATGGGCAATTGATCATGGTGCTTGGGGACATGCTTTATTTCTTGCATCTAAAATGGATG
AAAGAACTCATAACAATATAATGTTACGTTTTGCGAATAGCATTCCACATAATGATCCTTTGCA
GACCCTATATCAGCTAATGTCAGGCCATGTACCTCAAGCCTCCACTTGTTGTGCTGATAAGAAA
TGGTCCGATTGGAGGCCACATTTGGCTATGATTCTTGGAAATCCAACTGGAAATACCAAGCTGG
ACAGGAAGGCAATAATCAAACTTGGCGATTCCCTATTTTCTCGCGGTAGGCTTTTCGCTTCGCA
TTTCTGTTATGTTACTGCACAAGCTGAATTCACAAGCTATGATCAAGAAGCAAAATTTGTATTA
TTAGGATCAAACCCTAATCAAGAATTTACACAGTTTGCAAGTTGTAGAGCAATTATGTTAACAA
TGTGCTACGAATATGGATTAAAATTACGACAAGTAAATGCTAATATTCCTTCACTTCAGTTATA
CAAGTTAATTATGGCGACTCGTCTAATTGATAGTGGAAAAAATAGAAATGCATTACAGTATTGC
GAAATGGTTGCAAATGAAGCTGTCCGAAATGAATGTTGTGAAAGGCCACTAATTGCTTGTGTTA
TTGATTTATCATCTAAACTAAAAATGTTAGATCCAGCCTTAGCACTGACTGGTGATGTTGAAAC
CGATCCAGATTGGCTAGCCAAGTTAAAGAACTTTTATGATAATCTTCCTGAGGACTATGATGCT
GGATTAGCAATGAGACACGGTGTTTCTTCATCAACTGTTTCTGAAGTTGGCCAAGAAATTATTC
CTTGTCAACAAGAAAAACTTGAAACCAACGAGGAAATGGTGAATACTTACAGCAACGAACCATC
AATAAATAAAGTTGAATATTCTTTGCCACCACCTCAGCCTATACACTTACCTTTACAGCCTCCA
CTCTCTGGGCAGATGTCACTGCCACCTGAATTAGTTCACCAAGAGGAGCAACAGCAAACATATC
AACCAAGTTCTCTACCTCCTCTTGGACCGAATACATACTCTGCCGAACCATTTCCTCAACAGTC
TGCAGTAGATCCTTATTGGAGTTCTAGTAATGGTATTCCAACATCAAATTCCTACTCAAGACAT
GAAAATGTTGAATCTGTTTCTAGTTCGAAAAATAATTTTTTTAAAGCTAGTGAAGAACAGCTAA
AGCTTAATGATATTAGTAAGGAAAGTAACCGAAATAATAAAATATCAAATGATAACAAAGCTAC
TAAAAATGATGATGCTCAGCAGAATGCAGGATGGTTTGGAGGAATTTGGGAAAAATTATCCATT
CGACCAAAAAATCAAATGCGCCTTCCTGATGATAATAACCCATCGATTATTTGGGATGAAAAGA
AGAAGAAATGGGTAAATTTAGAAAGCGATGACGATGGGCAGCAAACAGTGAAACCACCGCCAAG
AATGGCGGAAACTGTTAATAAACCTCAGCAACTTTCAAATGAAATCACCTCATCAGCTGCACCA
CTACCCACTGGAAACAAATATAAAATTCAAAAAGGCAAACTGATGAAATCAAATTATGTTAATA
TCATGGGCTCATCTAGTACAAGTCAATCGAGTACAGTTCGCCCTATTGATGGATTATTCCCAAC
TCCTGCACAGACAAGCAACACTAATTTCTTTGTACCACCTCCTGCCGAGGGAAATGAATATCCT

CCAGTTGATTTCATTAATCCTGGAGCTATGGCTGGAAACAATCAACAAGCTGAAGGAACAACGG
AAATCAAGCAGGATTTCCCGCAAGGTATAACAGCTCCAACAATGTATAATCCAGTCAGATACCC
AATTAGCAATGATTGTTGATATTAAAAATATGTACCTAATTTTTGTTCATTTTTAATGTAAATA
TATTTTCTTGGTGCATTTATTGTAAATTTGTTTTGGAGTAACGAAATTGATACTTATATATGTA
AATGTAAACTTAAATTAAAATAGCTAAGTGATTCACTCATAAATCGTGATTTATTTTTATGTTC
AAAAAAGGCAAAAATTTTTTTCTAGAAATGGGTCTTTTTTTTAACATTAGGAAAAGACATTATT
GGTGATTTTATGATTTTATTTTAAATTCGTTTCTAATTAAATTACTTAGTCGTCATGGCTTATT
TGTGCAATGTTATTAAGGGAAATTGTATATTTGATATATATTGGCGTATAATTTTTATTAATTT
CCGCACAGTATATATTTATTAGGTGATATTTATAAAATCAAATTGGTTGCAGTTCTTGTTGTAT
AAATATATATATATATATATATAATATCATATTGTCACTAAAAATATATTGTAAACGTTTTAAT
ATATATACATAAAGTATTATTTAAGGATGATATTAGCTTGTTTTGTTTTGAACCAAAATGCATT
GAATTCAATACTGATTTGTGGTAAGAAGTTAAGGTATTGTGTTTACCCTGCTCTATTTAATGTT
CAAAATTAAATATGGATTGTGATTATTTAACAATGCATATTTCACAATACATATATATAAATCT
TCGGAATTAGTAACATTAAATACAGAATTTCTAAGAGCATATAATATTCTAAAATAAAATGG

- **Protein sequence:**

>*Rhodnius prolixus* Sec16

MNHSEQDPWTWGYEDSTVEDIQHNEWNAWNTCTPLTSDNKKNKDNSNKVKHPPNQGSEY
FDTFNLRNTSQNVQHNWTSGDAMQPNYANFVSHSNTSNYFNNNCQTDVENKEVAPPDNQ
YNRNSHQPPLPEDSKRDRHIQEQMTVPTFNQKKTTPIAAPLLGPSFGSKQFDPQDNMFQ
QPVKHPNSMSSAVANVSYNPVGQHYSTTTPYSKEQSFYFPSSLSHEGATGFQAHSNSLS
DLPSRQQNESNNSLSGPQSLPPFHSSSVSSLSNNSTDHIIQHSSSTSSTISDAFHTEKS
RHAHAVLPLNDNSSQNKHQQMRDLSQTGKMKSHFSQQNVSSKEQASVFSSAVADKDVAS
NRHEFSTHSSSLVDLAEDVSNFYHPSKQEIKEGDQTQLQQQLDFHGSHKSEPPQVSNQM
SAFKPSLARPPLIHQMPVSDSQQISVSQHLPVLQQPPFPQSSADPQHRSLFHHVPASQL
HHVPQQHTSQQLAVNQSLPTVQQSPISQHLPVSQGPTIDQHTPVSQNLPVSQDQNIVHH
PTISQHLPVSQDQNIVHHPSISQHLPVSLDQNIVHHPSISQHLPVSLDQNIVHHPTISQ
HLPVSQDQNIVHHPSISQHLPVSQDQNIVHYPPISQHLPLAQHQSVAQQPSNSLSHHLL
VSQQPLITADATIPHQSLSSLQNSVSQHVMPQSHPHSQQTPVSHYLMASQQPSGPQQPS
TSSLPVPTSQQSQISQESPDFLSSPSVQQTQVSQQLTISQQSQDSNNVQENPDTKNFFS
NLCQRDDHPQEIIMPDSQKIETNNLKLLQNVSVRNFNSISVDDSQISQVDLLPNSQQRD
KDTTLITQNSNNSLTELRKEATETVQLDNDTSNFNANTSRNQMSFNFQNVHPVASSSPV
PSHNIPDNQERLPENHELPDNTENLHLDMKQLTLKREVESDSQIHCQVDRNQYLETGHL
SGSKDHILSDNSPNFEAVDEGEAPPPGLHRLVTGQGCETRGPKTISDHQLLMVGADSTI

VDDHRQSSLSSSTVGSVSDNRNAQNGPPVGESEVHTRTGRLVQGQSIGDDTDGVREVPG
EATPFQNRIVLGQMGRGITPPLVQDNSTTRGDNSREREIVGRMVLGERYDDRDLTQNVN
SQDDFRQSHRSLRRSRRGESSYEDEEHDYPSDRDRRVDEYKRRGDDRVRHRNRREHRSP
EYRSDEEFEDRRGFSRMGRSERRPREFSPDMSYYNNSYYRDDHRRHRRYDHYRDDYDDY
YYRENRSRPSSRTGSDYRRMNMDYYSGRVNARPMFYSDLVGAIPYNPRAPEEYFEAMRR
LDPMGYAAWYNQYMNSRYNVQQSQSNYNNDRASVHSGQSSTNNQRPNVETPQAGVGEEI
GRDEEEFVPATAHIKGIIDNYGRLVVIDPNYSMDSKRRNINIYQISKLQSDPDLDEFLE
SPGPFIPGVTHRNTVLQYLKRISERSTKSSEKLLYDLVHLSVKSNGELNGLDVADLLME
SYKKSQSTEAPNLSEPLVEQISSTDALTKFRELLQQGNKTEALEWAIDHGAWGHALFLA
SKMDERTHNNIMLRFANSIPHNDPLQTLYQLMSGHVPQASTCCADKKWSDWRPHLAMIL
GNPTGNTKLDRKAIIKLGDSLFSRGRLFASHFCYVTAQAEFTSYDQEAKFVLLGSNPNQ
EFTQFASCRAIMLTMCYEYGLKLRQVNANIPSLQLYKLIMATRLIDSGKNRNALQYCEM
VANEAVRNECCERPLIACVIDLSSKLKMLDPALALTGDVETDPDWLAKLKNFYDNLPED
YDAGLAMRHGVSSSTVSEVGQEIIPCQQEKLETNEEMVNTYSNEPSINKVEYSLPPPQP
IHLPLQPPLSGQMSLPPELVHQEEQQQTYQPSSLPPLGPNTYSAEPFPQQSAVDPYWSS
SNGIPTSNSYSRHENVESVSSSKNNFFKASEEQLKLNDISKESNRNNKISNDNKATKND
DAQQNAGWFGGIWEKLSIRPKNQMRLPDDNNPSIIWDEKKKKWVNLESDDDGQQTVKPP
PRMAETVNKPQQLSNEITSSAAPLPTGNKYKIQKGKLMKSNYVNIMGSSSTSQSSTVRP
IDGLFPTPAQTSNTNFFVPPPAEGNEYPPVDFINPGAMAGNNQQAEGTTEIKQDFPQGI
TAPTMYNPVRYPISNDC
